# Shared and Divergent Transcriptional Programs of Oligodendrocyte Differentiation Across Vertebrate Species Revealed by scRNA-seq Analysis

**DOI:** 10.3390/ijms27104283

**Published:** 2026-05-11

**Authors:** Tery Yun, Junhee Park, Myungin Baek

**Affiliations:** Department of Brain Sciences, Daegu Gyeongbuk Institute of Science and Technology (DGIST), Daegu 42988, Republic of Korea; tery0520@dgist.ac.kr (T.Y.); pjhee1224@dgist.ac.kr (J.P.)

**Keywords:** oligodendrocyte, cross-species comparison, scRNA-seq, spinal cord, vertebrate evolution, aquatic–terrestrial divergence

## Abstract

A myelination is essential for neural function in the vertebrate central nervous system, yet the molecular details of how the oligodendrocyte differentiation program has evolved remain poorly understood. Here, we performed a cross-species single-cell transcriptomic analysis of oligodendrocyte lineage cells in the spinal cord of five vertebrate species: fugu, mudskipper, chicken, mouse, and human. Pseudotime trajectory analysis revealed a shared oligodendrocyte progenitor cell (OPC) to committed oligodendrocyte precursor (COP) to myelin-forming oligodendrocyte (MOL) differentiation trajectory across all species, and CAME-based cross-species mapping confirmed the homology of OPC and MOL identities, while COP showed reduced mapping in teleosts compared with amniotes. Among stage-specific DEGs, highly shared genes (≥4 species) were organized into four co-expression modules encompassing cell projection organization, myelination, synapse assembly, and ribonucleoprotein biogenesis, with evolutionary core genes (all 5 species) enriched for oligodendrocyte differentiation and Wnt signaling. Strikingly, amniote-exclusive genes were enriched for synaptic vesicle transport, cell projection organization, predominantly at the OPC stage. This asymmetry indicates that amniotes have expanded the oligodendrocyte differentiation program at the progenitor stage, potentially linked to the myelination demands of terrestrial locomotor circuits. Our findings provide insights into how the oligodendrocyte differentiation program has been shaped by both deep evolutionary conservation and lineage-specific adaptation.

## 1. Introduction

Myelination is a fundamental feature of the vertebrate nervous system that enables rapid and energy-efficient saltatory conduction of nerve impulses along axons [1]. In the central nervous system (CNS), myelin is produced by oligodendrocytes, specialized glial cells that extend multiple processes to ensheath axonal segments with a lipid-rich, multilayered membrane [2]. The formation and maintenance of myelin are essential for normal neural circuit function, and disruption of myelination underlies a range of neurological disorders, most notably multiple sclerosis (MS), in which autoimmune-mediated demyelination leads to progressive neurological disability [3].

Oligodendrocytes arise from OPCs through a tightly regulated differentiation process. OPCs are proliferative progenitors that are widely distributed throughout the CNS and persist into adulthood [4]. Upon receipt of appropriate signals, OPCs exit the cell cycle and undergo terminal differentiation through an intermediate COP state, ultimately maturing into MOL [5,6]. This differentiation program is governed by a complex regulatory network involving transcription factors (TFs) such as OLIG2, SOX10, NKX2-2, and MYRF, which orchestrate the sequential activation of myelin gene expression [7,8]. While the molecular mechanisms controlling oligodendrocyte differentiation have been extensively characterized in mice and, to a lesser extent, in humans, comparatively little is known about whether these mechanisms are conserved across the broader vertebrate phylogeny.

Myelination is thought to have evolved in the common ancestor of jawed vertebrates (gnathostomes) approximately 450 million years ago [9]. Although compact myelin is present across all major jawed vertebrate lineages—including cartilaginous fish, ray-finned fish, amphibians, reptiles, birds, and mammals—the molecular basis of myelination shows both conserved and divergent features. For instance, major myelin structural proteins such as MBP and proteolipid family proteins are conserved across vertebrates, yet their relative abundance and post-translational modification patterns differ across lineages [10,11,12]. Whether the upstream transcriptional program that drives oligodendrocyte differentiation and initiates myelination is similarly conserved remains an open question with important implications for both evolutionary biology and translational medicine.

Recent advances in scRNA-seq have transformed our ability to characterize cell type diversity and developmental trajectories at unprecedented resolution [13]. Cross-species scRNA-seq comparisons have provided valuable insights into the evolutionary conservation and divergence of cell types across organ systems, including the brain [14,15], immune system [16], and germline [17]. These studies have revealed that core transcriptional programs defining major cell types are often deeply conserved, while species-specific transcriptional variation contributes to phenotypic diversity. However, systematic cross-species comparisons of the oligodendrocyte lineage at single-cell resolution have not been performed, leaving a significant gap in our understanding of how the myelination program has evolved.

The spinal cord represents an ideal organ for such comparative analysis. As myelination in the spinal cord is essential for the proper function of these circuits, and oligodendrocyte lineage cells are consistently present in the spinal cords of all jawed vertebrates studied to date. Importantly, the transition from aquatic to terrestrial life imposed new demands on spinal cord circuitry, including the coordination of limb-based locomotion and weight-bearing posture, which required substantial remodeling of motor and sensory circuits [18]. Whether these ecological transitions were accompanied by changes in the myelination program that supports spinal cord circuit function has not been explored.

In this study, we performed a cross-species scRNA-seq analysis of oligodendrocyte lineage cells in the spinal cord of five vertebrate species: fugu (*Takifugu obscurus* X *Takifugu rubripes*), mudskipper (*Periophthalmus magnuspinnatus*), chicken (*Gallus gallus*), mouse (*Mus musculus*), and human (*Homo sapiens*). These species were selected to represent the major jawed vertebrate lineages, encompassing two teleost fishes, one avian species, and two mammals, and to span the aquatic–terrestrial divide, including the mudskipper, an amphibious fish capable of terrestrial locomotion on mudflats which occupies a unique ecological position at the aquatic–terrestrial interface [19]. We hypothesized that the oligodendrocyte differentiation program comprises both a deeply conserved core maintained across vertebrate lineages and lineage-specific elaborations that reflect the distinct ecological and locomotor demands of aquatic and terrestrial environments. By integrating phylogenomic analysis, pseudotime trajectory reconstruction, cross-species cell type mapping, and conserved gene module identification, we aimed to determine the extent to which the oligodendrocyte differentiation program is shared across vertebrate evolution. Specifically, we sought to identify both the core molecular programs maintained across all lineages and the lineage-specific elaborations that distinguish aquatic and terrestrial vertebrates.

## 2. Results

### 2.1. scRNA-seq Atlas of Five Vertebrate Spinal Cords

To investigate the evolutionary conservation of oligodendrocyte lineage differentiation programs across vertebrates, we constructed scRNA-seq atlases of spinal cords from five species spanning approximately 450 million years of vertebrate evolution. The species examined included two teleost fishes, fugu (*Takifugu obscurus X Takifugu rubripes*) and mudskipper (*Periophthalmus magnuspinnatus*), one avian species, chicken (*Gallus gallus*), and two mammals, mouse (*Mus musculus*) and human (*Homo sapiens*). These species represent the major jawed vertebrate lineages, encompassing both Actinopterygii (ray-finned fishes) and Sarcopterygii (lobe-finned fishes and tetrapods).

**Figure 1:**
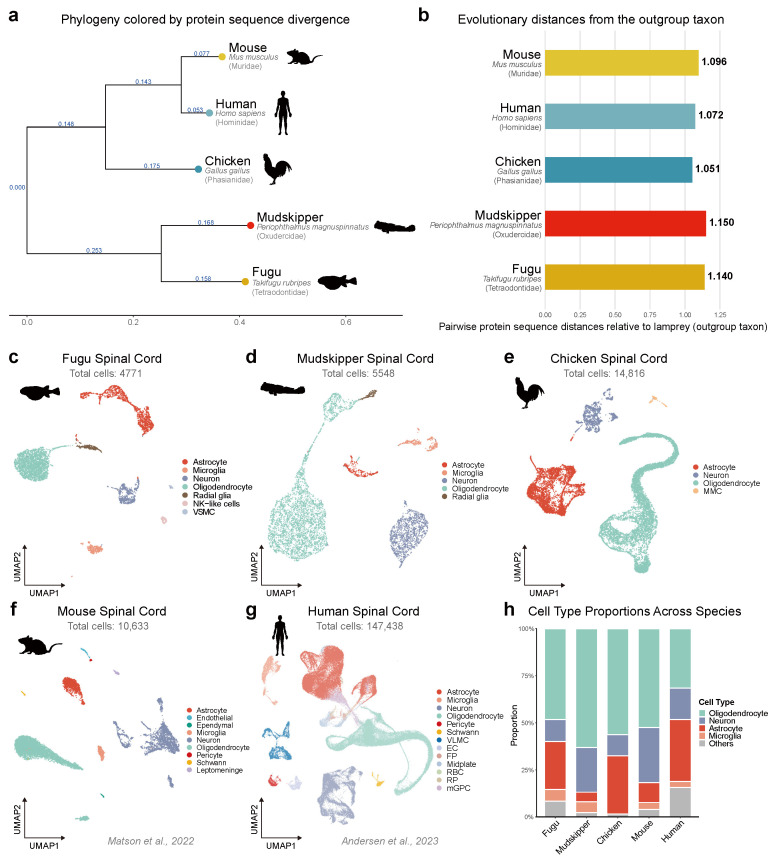
scRNA-seq atlas of five vertebrate spinal cords. (**a**) Maximum-likelihood phylogenetic tree of five vertebrate species constructed from concatenated single-copy orthologous protein sequences, with lamprey (*Petromyzon marinus*) as the outgroup. Branch lengths represent amino acid substitutions per site, and branches are colored by protein sequence divergence. (**b**) Pairwise protein sequence distances of each species relative to the lamprey outgroup. (**c**–**g**) UMAP visualizations of spinal cord single-cell transcriptomes for fugu (*T. obscurus* X *T. rubripes*; 4771 cells), mudskipper (*P. magnuspinnatus*; 5548 cells), chicken (*G. gallus*; 14,816 cells), mouse (*M. musculus*; 10,633 cells) [21], and human (*H. sapiens*; 147,438 cells) [22]. Cells are colored by annotated cell type. Abbreviations: VSMC, vascular smooth muscle cell (**a**); MMC, meningeal mesenchymal cell (**c**). Mouse and human UMAP plots were generated by reanalysis of publicly available datasets (GSE172167 [21] and GSE188516 [22], respectively). (**h**) Stacked bar plot showing the proportions of major cell types across five species.

A maximum-likelihood phylogenetic tree was constructed using concatenated single-copy orthologous protein sequences identified by OrthoFinder [20], with lamprey (*Petromyzon marinus*) as the outgroup (Figure 1a). The resulting topology recovered the expected vertebrate phylogeny, with a clear separation between teleost fishes and tetrapods. Pairwise protein sequence distances relative to lamprey showed comparable evolutionary divergence among the five species, ranging from 1.051 (chicken) to 1.150 (mudskipper), indicating that these species collectively capture a broad range of vertebrate molecular diversity (Figure 1b).

Spinal cord scRNA-seq data for fugu, mudskipper, and chicken were generated in this study, while publicly available datasets were used for mice [21] and humans [22]. Details of all samples are provided in Table 1, with a complete summary of the single-cell RNA sequencing datasets in Table S1. For the mouse dataset, only uninjured control samples were retained to ensure comparison of homeostatic transcriptional states. All datasets were reprocessed from count matrices using a unified analysis pipeline with Seurat [23] to minimize technical variation (see Methods). Quality control metrics, including UMI counts and detected gene counts per cell, were assessed across all species and cell types (Figure S1).

After quality control and unsupervised clustering, we obtained a total of 4771 cells for fugu, 5548 for mudskipper, 14,816 for chicken, 10,633 for mouse, and 147,438 for human (Figure 1c–g). Cell type annotation was performed based on the expression of canonical marker genes for each species (Figure S2). Major cell types, including neurons, astrocytes, oligodendrocytes, and microglia, were consistently identified across all five species. In the two teleost fishes, radial glia were identified as a distinct population expressing glial progenitor markers such as *Sox2*, *Vim*, and *Slc1a2*, consistent with the retention of radial glia as neural stem cells in the adult fish central nervous system [25].

Notably, oligodendrocyte lineage cells constituted the largest cell type proportion in all five species, ranging from 31.7% in humans to 63.1% in mudskipper (Figure 1h), with consistent compositions observed between biological replicates within each species (Figure S3). This consistent enrichment of oligodendrocyte lineage cells across evolutionarily divergent vertebrate spinal cords provided the cellular basis for subsequent cross-species comparative analysis of oligodendrocyte differentiation programs.

### 2.2. Conserved Pseudotime Trajectory of Oligodendrocyte Differentiation

**Figure 2:**
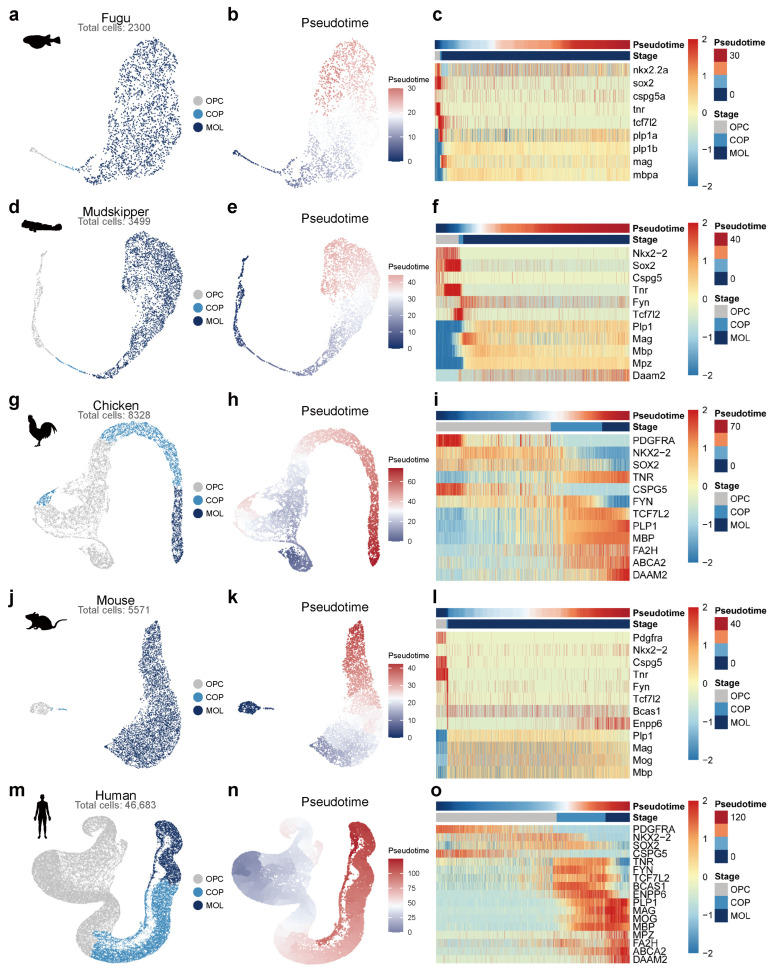
Pseudotime trajectory of oligodendrocyte differentiation across five species. For each species (fugu, (**a**–**c**); mudskipper, (**d**–**f**); chicken, (**g**–**i**); mouse, (**j**–**l**); human, (**m**–**o**), three panels are shown. (Left): UMAP of oligodendrocyte lineage cells colored by differentiation stage (OPC, light gray; COP, light blue; MOL, dark blue). (Middle): UMAP colored by pseudotime values inferred by Monocle3. (Right): Heatmaps showing the expression dynamics of representative marker genes along pseudotime, with cells ordered from OPC (left) to MOL (right). Conserved OPC markers (*Nkx2-2*, *Sox2*, *Cspg5*) are downregulated, while myelination genes (*Plp1*, *Mag*, *Mbp*) are upregulated along pseudotime in all species. Total oligodendrocyte lineage cell numbers: fugu, 2300; mudskipper, 3499; chicken, 8328; mouse, 5571; human, 46,683.

To characterize the oligodendrocyte differentiation trajectory in each species, we extracted oligodendrocyte lineage cells and performed pseudotime analysis using Monocle3 [26]. For each species, the trajectory root was objectively defined using an OPC root score calculated from known progenitor markers, including *Pdgfra*, *Nkx2-2*, *Sox2*, and *Cspg5* (Figure S4). The resulting pseudotime values increased monotonically from OPCs toward MOLs in all five species (Figure 2b,e,h,k,n).

Cells were classified into three differentiation stages—OPC, COP, and MOL—based on the crossover points of stage-specific gene module scores along the pseudotime axis (Figure S4). The OPC module included progenitor markers such as *Pdgfra*, *Nkx2-2*, *Sox2*, *Cspg5*, and *Tnr*; the COP module included transition markers such as *Fyn* and *Tcf7l2*; and the MOL module included myelination-associated genes such as *Plp1*, *Mag*, *Mbp*, and *Mog*. This marker-based staging approach ensured objective and reproducible classification across species.

Pseudotime heatmaps of representative marker genes revealed a conserved temporal expression pattern across all five species (Figure 2c,f,i,l,o). OPC-associated genes, including *Nkx2-2*, *Sox2*, and *Cspg5*, were highly expressed at the beginning of pseudotime and progressively downregulated. Conversely, myelination genes such as *Plp1*, *Mag*, and *Mbp* exhibited increasing expression along pseudotime, consistent with their role in terminal differentiation. The intermediate marker *Tcf7l2*, a TF implicated in oligodendrocyte lineage progression [27,28], displayed transient upregulation at the COP stage across species (Figure S5). These results demonstrate that the fundamental transcriptional program underlying the OPC-to-MOL differentiation trajectory is conserved from teleost fishes to mammals.

### 2.3. Cross-Species Cell Type Correspondence and Divergence of Oligodendrocyte Differentiation Programs

**Figure 3:**
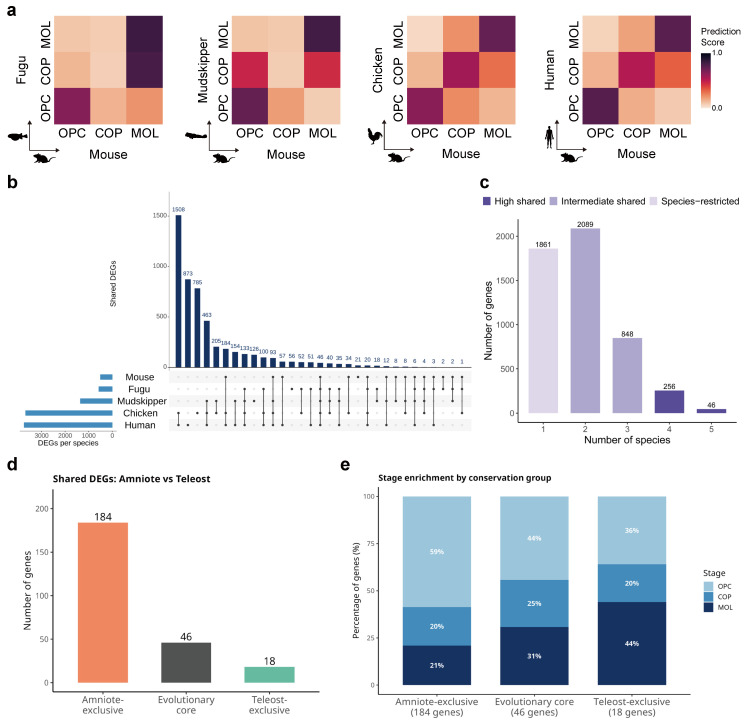
Cross-species cell type correspondence and divergence of oligodendrocyte differentiation programs along the aquatic–terrestrial axis. (**a**) CAME prediction score heatmaps for cross-species cell type mapping. Each heatmap shows the prediction score between oligodendrocyte subtypes of each query species (fugu, mudskipper, chicken, and human) and the mouse reference. Prediction scores are color-coded. (**b**) UpSet plot showing the intersection of stage-specific DEGs across five species. Bars represent the number of genes shared by each species combination. (**c**) Bar plot showing the number of DEGs classified as high-shared (≥4 species, dark blue), intermediate-shared (2–3 species, medium blue), and species-restricted (1 species, light blue). (**d**) Bar plot comparing the number of amniote-exclusive DEGs (184 genes; shared by chicken, mouse, and human but absent in both teleosts), evolutionary core DEGs (46 genes; shared by all five species), and teleost-exclusive DEGs (18 genes; shared by fugu and mudskipper but absent in all amniotes). (**e**) Stacked bar plot showing the stage enrichment of DEGs in each conservation group.

To quantitatively assess the cross-species correspondence of oligodendrocyte differentiation stages, we applied CAME (Cell-type Assignment and gene Module Extraction), a heterogeneous graph neural network-based method for cross-species cell-type assignment [29]. Using mouse as a reference species, CAME predicted the cell type identity of oligodendrocyte lineage cells from the four query species (fugu, mudskipper, chicken, and human). The resulting prediction score heatmaps showed strong diagonal patterns for OPC and MOL across all species pairs, with OPCs consistently mapping to mouse OPCs and MOLs to mouse MOLs (Figure 3a). For COP, chicken and human showed clear mapping to mouse COP, whereas fugu and mudskipper COPs displayed prediction scores distributed between mouse OPC and MOL. Downsampling analysis confirmed that this difference was not attributable to total cell number, as random downsampling of chicken and human to match the smallest dataset (fugu; 2300 cells) preserved robust COP-to-COP mapping (Figure S6), suggesting that the reduced COP mapping in teleosts reflects biological differences in COP cell proportions rather than a technical artifact of dataset size.

To further examine the molecular basis of this conservation, we identified stage-specific DEGs in each species and performed cross-species comparison. An UpSet plot revealed complex patterns of gene sharing across species (Figure 3b). When categorized by the number of species sharing each gene, 302 genes were classified as high-shared (detected in ≥4 species), 2937 as intermediate-shared (2–3 species), and 1861 as species-restricted (1 species) (Figure 3c). Among the high-shared genes, 46 genes were detected in all five species and designated as evolutionary core genes.

Strikingly, the distribution of DEGs revealed a marked asymmetry along the aquatic–terrestrial axis. Although there are significant overlaps between amniotes and teleosts among DEGs, a total of 184 genes were shared exclusively among the three amniote species (chicken, mouse, and human) but absent from both teleosts, whereas only 18 genes were shared exclusively between the two teleost species (fugu and mudskipper) but absent from all amniotes (Figure 3d).

All 184 amniote-exclusive genes had identifiable one-to-one orthologs in both teleost genomes, and 172 (93.5%) were expressed in the teleost spinal cord, confirming that their absence from the teleost oligodendrocyte DEG list reflects biological differences in transcriptional regulation rather than genome annotation artifacts (Table S8). Analysis of stage enrichment showed that amniote-exclusive genes were predominantly associated with the OPC stage (59%), whereas teleost-exclusive genes showed a bias toward the MOL stage (44%) (Figure 3e). This asymmetry provides a molecular explanation for the reduced COP mapping observed in teleosts in the CAME analysis: the 184 amniote-exclusive genes, concentrated at the OPC stage, likely contribute to a more transcriptionally defined OPC-to-COP transition in amniotes, whereas the absence of these genes in teleosts results in a less distinct COP identity.

To assess the impact of developmental stage on these findings, we performed an independent validation by replacing the adult mouse dataset with postnatal (P2 + P11) mouse spinal cord data (SPLiT-seq) [24]. The postnatal mouse oligodendrocyte lineage showed a continuous OPC-to-COP-to-MOL trajectory on the UMAP, in contrast to the spatial separation observed in the adult mouse dataset (Figure S8a,b). All key cross-species metrics increased with the postnatal dataset: mouse DEGs (501 to 621), high-shared genes (302 to 327), evolutionary core genes (46 to 68), and amniote-exclusive genes (184 to 243) (Figure S8g–i). These results confirm that developmental stage affects DEG detection and that the adult mouse-based analysis provides a conservative estimate of cross-species gene sharing.

We also examined the stage-specific expression of TF genes across species. Heatmap analysis of OPC-, COP-, and MOL-enriched TF genes revealed a set of shared regulators alongside species-specific factors (Figure S10). Among OPC TF genes, *Egr1*, *Meis2*, *Myt1l*, *Sox5*, and *Sox6* were identified as OPC-enriched TFs across ≥4 species. COP-enriched TF genes included *Skil*, *Tcf7l2*, and *Zfp516* as broadly shared factors. *Myrf*, the master regulator of myelination [8], was identified as a MOL-enriched TF gene shared across ≥4 species, together with *Nkx6-2*, *Tcf12*, and *Zfp536*. Stage-specific DEG and TF heatmaps are shown in Figures S9 and S10, with the complete gene lists provided in Tables S4 and S5, respectively.

### 2.4. Conserved Gene Modules Underlying Oligodendrocyte Differentiation

**Figure 4:**
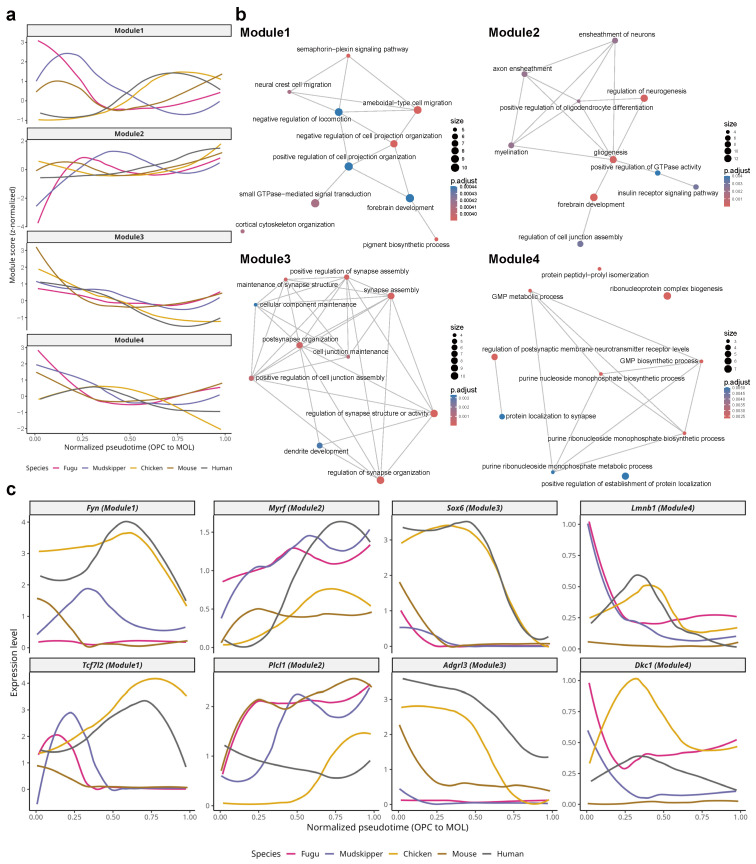
Conserved gene modules underlying oligodendrocyte differentiation. (**a**) Module score dynamics along normalized pseudotime (0 = OPC, 1 = MOL) for four co-expression modules identified from 302 high-shared genes (detected in ≥4 species; Table S3). Each line represents one species. Module 1 (93 genes): early declining expression; Module 2 (107 genes): progressive upregulation toward MOL; Module 3 (52 genes): transient peak expression; Module 4 (50 genes): early transient expression. (**b**) Gene Ontology enrichment map networks for each module. Node size represents the number of enriched genes; node color represents adjusted *p*-value. Module 1: cell projection organization and semaphorin-plexin signaling; Module 2: myelination and axon ensheathment; Module 3: synapse assembly and organization; Module 4: ribonucleoprotein complex biogenesis. (**c**) Expression dynamics of representative genes from each module along normalized pseudotime across five species. Module 1: *Fyn* and *Tcf7l2*; Module 2: *Myrf* and *Plcl1*; Module 3: *Sox6* and *Adgrl3*; Module 4: *Lmnb1* and *Dkc1*.

To identify the shared molecular programs driving oligodendrocyte differentiation, we performed co-expression module analysis on the 302 high-shared genes (detected in ≥4 species; Table S3), following the cross-species comparative framework described by [17]. Pseudotime values were normalized to a 0–1 scale (OPC to MOL) across species, and gene expression dynamics were clustered to identify four distinct modules with characteristic temporal profiles (Figure 4a).

Module 1 (93 genes) exhibited high expression in early pseudotime (OPC stage) with progressive downregulation toward MOL. Gene Ontology (GO) enrichment analysis revealed that Module 1 was significantly enriched for terms related to cell projection organization, semaphorin-plexin signaling, small GTPase-mediated signal transduction, and amoeboidal-type cell migration (Figure 4b and Figure S11a; Table S6). Representative genes of this module included *Fyn*, a Src family kinase involved in oligodendrocyte process extension, and *Tcf7l2*, a TF gene that regulates the transition from OPC to differentiating oligodendrocyte (Figure 4c). These results suggest that conserved signaling pathways governing cell morphology and migration are active during the early stages of oligodendrocyte specification.

Module 2 (107 genes) showed progressive upregulation from COP to MOL, reaching peak expression in mature oligodendrocytes. This module was enriched for myelination, axon ensheathment, ensheathment of neurons, gliogenesis, and regulation of oligodendrocyte differentiation (Figure 4b and Figure S11b; Table S6). Representative genes included *Myrf*, the master transcriptional regulator of the myelination program [8], and *Plcl1*, a phospholipase C-like protein that has been associated with lipid signaling in the nervous system (Figure 4c). The enrichment of myelination-related terms in this module confirms that the core myelin biosynthesis program has been evolutionarily conserved across the five vertebrate species examined.

Module 3 (52 genes) displayed a transient expression pattern, peaking at the COP-to-MOL transition before declining. Unexpectedly, this module was enriched for synapse-related terms, including synapse assembly, regulation of synapse organization, postsynapse organization, and maintenance of synapse structure (Figure 4b and Figure S11c; Table S6). Representative genes included *Sox6*, a TF gene involved in oligodendrocyte maturation, and *Adgrl3*, an adhesion G protein-coupled receptor gene (Figure 4c). The enrichment of synaptic terms in oligodendrocyte differentiation may reflect the role of neuron–oligodendrocyte communication in activity-dependent myelination, which requires oligodendrocytes to sense and respond to synaptic activity.

Module 4 (50 genes) showed early transient expression in OPCs, declining rapidly during differentiation. This module was enriched for ribonucleoprotein complex biogenesis, purine nucleoside monophosphate biosynthetic processes, and GMP metabolic processes (Figure 4b and Figure S11d; Table S6). Representative genes included *Lmnb1*, a nuclear lamin gene associated with oligodendrocyte nuclear architecture, and *Dkc1*, a pseudouridine synthase gene involved in ribosome biogenesis (Figure 4c). The early expression of this module suggests that active ribosome biogenesis and nucleotide metabolism are required during the proliferative OPC phase to support the high biosynthetic demands of subsequent differentiation and myelination.

Detailed expression patterns of all genes within each module are shown in Figure S11, and individual GO biological process enrichment for each module is presented in Figure S12 (full results in Table S6). Collectively, these four gene modules delineate a shared molecular framework for oligodendrocyte differentiation that has been maintained across ~450 million years of vertebrate evolution, encompassing progenitor signaling (Module 1), core myelination (Module 2), neuron–glia communication (Module 3), and biosynthetic capacity (Module 4).

### 2.5. Evolutionary Core Program and Amniote-Specific Expansion

**Figure 5:**
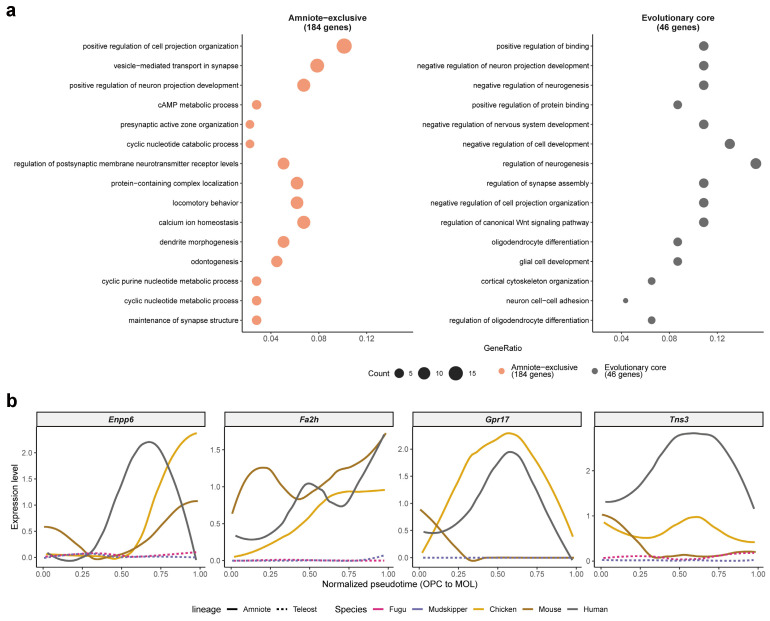
Evolutionary core program and amniote-specific expansion of the oligodendrocyte differentiation program. (**a**) Comparative Gene Ontology (GO) biological process enrichment analysis of evolutionary core genes (46 genes shared by all five species; gray) and amniote-exclusive genes (184 genes shared by chicken, mouse, and human but absent in both teleosts; orange). Dot size represents the number of genes associated with each term. (**b**) Pseudotime expression dynamics of four representative amniote-exclusive genes across five species. Solid lines indicate amniote species (chicken, mouse, human); dotted lines indicate teleost species (fugu, mudskipper). *Tns3* (committed OPC marker gene) and *Gpr17* (OPC differentiation timer gene) peak at the OPC-to-COP transition, while *Enpp6* (new oligodendrocyte marker gene) and *Fa2h* (myelin lipid 2-hydroxylase gene) peak at later differentiation stages.

To further dissect the hierarchical organization of the oligodendrocyte differentiation program, we performed functional enrichment analysis on the 46 evolutionary core genes (shared across all five species) and the 184 amniote-exclusive genes separately (Figure 5a). GO enrichment analysis revealed that the evolutionary core genes were enriched for fundamental oligodendrocyte-related functions, including regulation of neurogenesis, oligodendrocyte differentiation, glial cell development, and regulation of canonical Wnt signaling pathway (Figure 5a and Figure S13b). These results indicate that the most deeply shared component of the differentiation program encompasses the core transcriptional regulatory machinery for oligodendrocyte fate determination and the Wnt–Tcf7l2 signaling axis.

In contrast, the 184 amniote-exclusive genes were enriched for functionally distinct categories, including positive regulation of cell projection organization, vesicle-mediated transport in synapse, locomotory behavior, presynaptic active zone organization, and dendrite morphogenesis (Figure 5a and Figure S13a). These terms indicate that amniotes have acquired an expanded set of genes involved in neuron–oligodendrocyte synaptic-like interactions and cellular morphological complexity, which are not shared with teleost fish.

To validate the amniote-exclusive expression pattern at the individual gene level, we examined the pseudotime dynamics of four representative genes: *Tns3*, a committed OPC marker [6]; *Gpr17*, an OPC differentiation timer [30]; *Enpp6*, a marker of newly formed oligodendrocytes [31]; and *Fa2h*, a myelin lipid 2-hydroxylase [32] (Figure 5b). All four genes displayed prominent expression dynamics along the differentiation trajectory in amniote species (chicken, mouse, and human) but showed minimal or absent expression in both teleost species (fugu and mudskipper). *Tns3* and *Gpr17* peaked at the OPC-to-COP transition, while *Enpp6* and *Fa2h* peaked at later stages, collectively demonstrating that the amniote-specific elaboration spans the entire differentiation trajectory from early progenitor commitment to terminal maturation.

## 3. Discussion

In this study, we performed a comprehensive cross-species scRNA-seq analysis of oligodendrocyte lineage cells across five vertebrate species spanning the major vertebrate lineages, from teleost fishes to mammals. By integrating phylogenomics, pseudotime trajectory analysis, cross-species cell type mapping, and conserved gene module identification, we revealed that the oligodendrocyte differentiation program is organized as a hierarchical architecture: an ancient core shared across all vertebrates, supplemented by an expanded set of genes in the amniote lineage. Our analysis identified 302 high-shared genes organized into four functionally distinct modules, 46 evolutionary core genes representing the most deeply maintained differentiation machinery, and 184 amniote-exclusive genes enriched for synaptic and morphological complexity programs, collectively demonstrating both the conservation and diversification of the myelination program across vertebrate evolution.

### 3.1. A Shared Oligodendrocyte Differentiation Trajectory Across Vertebrates

A central finding of this study is that the OPC-to-MOL differentiation trajectory is remarkably consistent across all five species examined, despite substantial differences in evolutionary distance and developmental stage. CAME-based cross-species mapping confirmed that OPC and MOL populations defined independently in each species corresponded to their mouse counterparts with high prediction scores, providing independent validation that these cell states represent genuinely homologous transcriptional identities rather than artifacts of annotation. However, COP cells in the two teleost species showed diffuse mapping between mouse OPC and MOL rather than clear assignment to mouse COP, in contrast to the robust COP self-mapping observed in chicken and human. This lineage-dependent difference in COP identity may reflect the substantial asymmetry in differentiation-associated gene repertoires between amniote and teleost species, as discussed in detail below. These observations suggest that while the endpoints of the differentiation trajectory (OPC and MOL) are shared across all vertebrates, the intermediate COP state has been further elaborated in amniotes. Notably, in the adult mouse dataset, OPC/COP and MOL appeared spatially separated on the UMAP, which reflects the predominance of terminally differentiated MOLs in the adult spinal cord and the reduced cell number after exclusion of injured samples from the original dataset [21]. An independent analysis using postnatal mouse spinal cord data (P2 + P11) [24] confirmed a continuous OPC-to-COP-to-MOL trajectory (Figure S8d,e), indicating that this separation reflects the cellular composition of the mature spinal cord rather than a biological discontinuity in the differentiation program. The identification of shared stage-specific TFs further supports this conclusion: MYRF as a MOL-enriched TF is consistent with its established role as the master regulator of the myelination program [8], TCF7L2 at the COP stage confirms its role as a key transitional regulator [27,28], and shared OPC TFs such as EGR1, SOX5, and SOX6 point to a deeply maintained progenitor program. The maintenance of this trajectory across species separated by over 450 million years of evolution suggests that the core regulatory logic of oligodendrocyte differentiation was established early in vertebrate evolution, likely coinciding with the emergence of myelination in the common ancestor of jawed vertebrates [9].

### 3.2. Four Shared Molecular Programs Underlying Differentiation

The co-expression module analysis of 302 high-shared genes revealed four modules with distinct temporal dynamics and biological functions. Module 2, enriched for myelination and axon ensheathment, included well-characterized regulators such as *Myrf* and lipid metabolism genes critical for the biosynthesis of myelin membranes [2]. Module 1, enriched for cell projection organization and semaphorin-plexin signaling, likely reflects the dynamic morphological changes that OPCs undergo as they extend processes toward target axons. Module 3, unexpectedly enriched for synapse-related terms, displayed transient expression during the COP-to-MOL transition, consistent with the emerging concept of activity-dependent myelination in which oligodendrocyte lineage cells sense neuronal activity through neurotransmitter signaling and synaptic-like axo-glial interactions [33,34,35]. Module 4, enriched for ribonucleoprotein complex biogenesis, reflects the substantial translational demands placed on proliferating OPCs [1]. Together, these four modules suggest a shared molecular logic for oligodendrocyte differentiation that has been maintained as an integrated unit across the major vertebrate lineages.

### 3.3. An Evolutionary Core Program for Oligodendrocyte Fate Determination

Among the 302 high-shared genes, 46 evolutionary core genes detected in all five species were enriched for oligodendrocyte differentiation, glial cell development, and regulation of canonical Wnt signaling, representing the most fundamental layer of the differentiation program. The enrichment of Wnt signaling is notable because the Wnt–Tcf7l2 axis is a well-characterized regulator of oligodendrocyte differentiation timing [27,28], and our data suggest that this signaling pathway has been a conserved component of the oligodendrocyte differentiation program since the last common ancestor of teleost and tetrapods. The evolutionary core genes were distributed across all four temporal modules, indicating that the most deeply maintained program is not restricted to a particular differentiation stage but rather encompasses regulatory components spanning the entire OPC-to-MOL trajectory.

### 3.4. Divergence of the Differentiation Program Along the Aquatic–Terrestrial Axis

A key finding of this study is the marked asymmetry in DEG sharing between amniote and teleost species. The three amniote species shared 184 exclusive DEGs that were absent from both teleosts, whereas only 18 genes were exclusive to the two teleost species—an approximately 10-fold difference. This asymmetry suggests that the oligodendrocyte differentiation program has undergone substantial elaboration in the amniote lineage since the divergence of fish species. As shown in the validation analysis (Section 2.3), replacing the adult mouse dataset with postnatal (P2 + P11) mouse spinal cord data resulted in increases across all key metrics, confirming that developmental stage affects DEG detection. This result indicates that our main analysis using adult mouse data provides a conservative estimate and that the amniote-exclusive expansion is likely more extensive than reported here. Importantly, the detection of the amniote-exclusive program in both adult and postnatal mouse datasets demonstrates that this program is not restricted to developmental stages but is maintained throughout the lifespan of the oligodendrocyte lineage.

The amniote-exclusive genes were enriched for synaptic vesicle transport, cell projection organization, presynaptic active zone organization, and locomotory behavior, and were predominantly associated with the OPC stage (59%). This functional profile suggests that amniotes have expanded the repertoire of genes governing neuron–oligodendrocyte interactions and oligodendrocyte morphological complexity during the early stages of differentiation. The transition from aquatic to terrestrial locomotion is thought to have been accompanied by diversification of vertebrate appendage and spinal cord motor circuits in response to distinct biomechanical demands in water and on land [36,37]. Our data are consistent with the possibility that the elaboration of the oligodendrocyte differentiation program coevolved with the diversification of these circuits during vertebrate locomotor evolution.

This interpretation is further supported by the CAME analysis, in which teleost COP cells mapped diffusely between OPC and MOL, whereas amniote COPs displayed clear self-mapping. Downsampling analysis demonstrated that this difference was not attributable to total cell number, as random downsampling of chicken and human to match fugu cell numbers preserved robust COP mapping (Figure S6). Rather, the reduced COP mapping in teleosts likely reflects the low proportion of COP cells in these species, which may result from a more rapid OPC-to-MOL transition [38] combined with developmental stage differences, as the embryonic/fetal samples (chicken and human) capture a larger population of actively transitioning COP cells compared with the juvenile teleost datasets. The absence from the teleost transcriptome of 184 amniote-exclusive genes, many of which are OPC-enriched, likely contributes to a less transcriptionally defined COP intermediate in fish, in which the OPC-to-MOL transition may proceed more rapidly [38] or through a less molecularly distinct intermediate state. Representative amniote-exclusive genes include *Gpr17*, an OPC differentiation timer; [30]; Tns3, a committed OPC marker; Enpp6, a marker of newly formed oligodendrocytes [31]; and Fa2h, a myelin lipid 2-hydroxylase; [32]. *Gpr17* has been implicated in restraining premature differentiation of oligodendrocyte progenitor cells in both mouse and zebrafish [30,39]. Thus, the lack of Gpr17 expression in oligodendrocyte lineage may permit a more rapid transition from the progenitor cells to mature oligodendrocytes in mudskipper and fugu. However, its expression pattern and functional role in these fish species remain to be experimentally validated.

### 3.5. Implications for Demyelinating Diseases

The identification of a hierarchically organized oligodendrocyte differentiation program has implications for understanding demyelinating diseases such as multiple sclerosis (MS). The 46 evolutionary core genes, maintained across all five species spanning ~450 million years of divergence, represent the most fundamental and presumably indispensable components of the oligodendrocyte differentiation machinery. These genes, enriched for Wnt signaling and oligodendrocyte fate determination, may represent particularly attractive targets for remyelination strategies, as their deep evolutionary maintenance suggests essential and non-redundant roles in the differentiation process. The 184 amniote-exclusive genes, enriched for synaptic vesicle transport and cell projection organization, point to amniote-specific aspects of the myelination program that may be relevant to human-specific disease mechanisms. The evolutionary conservation of these pathways is consistent with the observation that demyelinating pathology can be modeled across diverse vertebrate species including zebrafish and rodents [40], while the amniote-specific elaborations identified in our study may in part explain why certain aspects of human MS pathology, particularly immune-mediated demyelination, are difficult to fully recapitulate in fish models [40].

### 3.6. Limitations

Several limitations of this study should be acknowledged. First, the datasets encompassed different developmental stages: fugu and mudskipper were sampled from juvenile individuals approximately one month post-hatching, chicken spinal cord was collected at embryonic days 10–11, human data were derived from gestational weeks 17–18, and mouse data were obtained from 12- to 30-week-old adults. Although oligodendrocyte lineage cells were identified in all species and displayed conserved differentiation trajectories, stage-dependent differences in cell maturity and transcriptional programs cannot be fully excluded. To assess the impact of this developmental variation in mice, we repeated the cross-species analysis using an independent early postnatal (P2 + P11) mouse spinal cord dataset (SPLiT-seq) [24]. All key metrics remained highly consistent (Figure S8), indicating that developmental stages, at least in mice, minimally affect DEG detection. Of note, the predominance of OPC-stage genes among the amniote-exclusive set should be interpreted with caution, as the embryonic and fetal stages of chicken and human datasets may contain a higher proportion of actively differentiating OPCs compared with the adult mouse. However, the requirement that amniote-exclusive genes be detected in all three amniote species, including the adult mouse, mitigates this concern, as does the observation that the juvenile teleost datasets—which are also expected to contain actively differentiating OPCs—did not share these genes. Additionally, the developmental stages of non-model species such as fugu and mudskipper are not as precisely characterized as those of model organisms. Although both were collected as juveniles approximately one month post-hatching, the exact stage of oligodendrocyte differentiation may differ between these species, potentially contributing to the variation in DEG detection and cross-species overlap patterns observed in the UpSet plot (Figure 3b). Relatedly, the reduced COP mapping observed in teleosts in the CAME analysis may partly reflect developmental stage-dependent differences in COP proportions: embryonic/fetal samples (chicken and human) contained a larger population of actively transitioning COP cells, whereas juvenile/adult samples (teleosts and mouse) were dominated by MOLs with few COPs. Downsampling analysis confirmed that total cell number did not account for this difference (Figure S6).

Second, for the newly generated datasets (fugu, mudskipper, and chicken), only the rostral spinal cord at the pectoral/wing level was sampled. Cell type composition is known to vary along the rostrocaudal axis of the spinal cord, and our results may not fully represent the cellular diversity of the entire spinal cord. The publicly available mouse and human datasets were derived from broader spinal cord regions, which may contribute to the observed differences in cell type proportions across species.

Third, the cross-species comparison relied on one-to-one ortholog mappings, which inherently excludes genes that have undergone duplication or loss in specific lineages. Teleost fishes, which experienced whole-genome duplication [41], may harbor additional paralogs with subfunctionalized roles in myelination that are not captured by this approach.

Fourth, the mouse dataset was generated using single-nucleus RNA-seq (snRNA-seq), whereas the in-house datasets (fugu, mudskipper, and chicken) were profiled using single-cell RNA-seq (scRNA-seq) and the human dataset using a combined scRNA-seq and snRNA-seq approach. The mouse dataset showed lower per-cell gene detection (median 434 genes per cell) compared with the scRNA-seq-based datasets (median 1450–3812 genes per cell; Table S9), which may reflect a combination of platform differences and the transcriptional characteristics of mature adult spinal cord cells. This lower gene detection inherently limits the number of detectable DEGs in mouse and reduces its pairwise overlap with other species, which likely contributes to the observation that human–chicken shared more DEGs than human–mouse despite the closer phylogenetic relationship between human and mouse.

Finally, the number of cells in the oligodendrocyte lineage varied considerably across species, ranging from 2300 cells in fugu to over 46,000 in humans. This variation may influence the statistical power to detect dynamically expressed genes in species with fewer cells, potentially underestimating the true degree of conservation.

## 4. Materials and Methods

### 4.1. Animals

All methods employed in this manuscript are reported in accordance with ARRIVE guidelines (https://arriveguidelines.org accessed on 1 April 2026). All experiments were approved by the Institutional Animal Care and Use Committee (IACUC) of the Daegu Gyeongbuk Institute of Science and Technology (DGIST) (Approval No: DGIST-IACUC-23052304-0002). All procedures were conducted in accordance with approved protocols and laboratory safety guidelines of DGIST.

### 4.2. Phylogenomic Analysis

Protein sequences from six vertebrate species were used for phylogenomic analysis: fugu (*Takifugu obscurus* X *Takifugu rubripes*), mudskipper (*Periophthalmus magnuspinnatus*), chicken (*Gallus gallus*), mouse (*Mus musculus*), human (*Homo sapiens*), and lamprey (*Petromyzon marinus*) as the outgroup. Proteome sequences for mudskipper, chicken, mouse, human, and lamprey were downloaded from Ensembl (release 115) [42]. Because a reference genome for the fugu species used in this study (*T. obscurus* X *T. rubripes*) was not available, the proteome of the closely related congeneric species *Takifugu rubripes* [43,44] was retrieved from Ensembl and used as a proxy. For each species, only the longest protein isoform per gene was retained before orthology inference.

Orthology analysis was performed using OrthoFinder (v2.5.5) [20] on the six-species proteome set. Orthogroups were inferred across all species, and 2215 single-copy orthogroups containing exactly one gene from each species were selected for downstream phylogenetic analysis. After alignment and trimming, 2213 orthologs were retained and concatenated into a supermatrix (1,244,390 amino acid sites; 251,905 parsimony-informative sites). For each single-copy orthogroup, protein sequences were aligned with MAFFT (v7.526) [45] and trimmed with trimAl (v1.5.rev1) [46] to remove poorly aligned regions. The resulting alignments were concatenated using AMAS (v1.0) to generate a supermatrix for phylogenetic inference. Maximum-likelihood phylogenetic analysis was performed using IQ-TREE (v3.0.1) [47], and branch support was assessed using bootstrap- and likelihood-based support measures. The best-fit substitution model was automatically selected using ModelFinder (integrated within IQ-TREE v3.0.1) [48] as implemented in IQ-TREE. The tree was rooted using lamprey as the outgroup. Lamprey was subsequently pruned from the displayed tree as no transcriptomic data were available for this species. Relative phylogenetic divergence among species was summarized by calculating pairwise protein sequence distances from the rooted tree, using lamprey as the reference lineage. The phylogeny and corresponding distance-based visualizations were generated in R (v4.4.3) using ggtree (v3.14.0) [49] and ggplot2 (v3.5.2) [50].

In addition to phylogenetic inference, OrthoFinder output was used to generate one-to-one ortholog mapping files between mouse and each of the remaining four species. These mappings were used to convert gene identifiers across species to mouse gene symbols for downstream cross-species transcriptomic comparisons.

### 4.3. Ortholog Identification

For cross-species transcriptomic comparison, one-to-one ortholog mappings between mouse and each of the remaining four species were derived from the OrthoFinder (v2.5.5) [20] output generated during the phylogenomic analysis. A custom Python script was used to extract pairwise ortholog relationships from the OrthoFinder results, converting protein identifiers back to gene-level identifiers using the original FASTA headers. For fugu, mudskipper, and chicken, mouse gene names were mapped to species-specific Ensembl gene identifiers, with Ensembl [42] version suffixes removed for compatibility with downstream single-cell expression matrices. For humans, mouse gene names were mapped to human gene names, as gene symbol-level matching was sufficient for cross-species comparison without requiring Ensembl identifiers. In all cases, only bidirectionally unique one-to-one ortholog pairs were retained to ensure unambiguous gene correspondence across species. The resulting ortholog pairs are summarized in Table S2. For CAME-based cross-species cell type mapping, the full ortholog mapping tables containing both one-to-one and one-to-many relationships were used as input, as CAME is designed to leverage non-unique homologous gene mappings to improve cell type assignment across distant species [29].

### 4.4. Cell-Sorting and scRNA-seq Library Preparation

The species, developmental stages, sample sizes, and sequencing platforms used in this study are summarized in Table 1. Spinal cords were dissected from juvenile fugu (*T. obscurus* X *T. rubripes*) and mudskipper (*P. magnuspinnatus*) approximately one month post-hatching, and from chicken (*G. gallus*) at embryonic days 10–11 (Hamburger–Hamilton stage 36–37). For all three species, the rostral spinal cord corresponding to the pectoral fin (fugu, mudskipper) or wing (chicken) level was used to ensure anatomical consistency across species. For each species, spinal cord tissue from multiple individuals was pooled per library to ensure sufficient cell yield. Cell dissociation was performed following an established protocol: briefly, spinal cord samples were incubated in a pronase solution for 40 min at room temperature, followed by mechanical trituration in 100 µL of artificial cerebrospinal fluid (ACSF) containing 1% FBS. The volume was then adjusted to 3 mL, and live cells were enriched by fluorescence-activated cell sorting (FACS; BD FACSAria III, BD Biosciences, San Jose, CA, USA) with propidium iodide (1 µg/mL) exclusion, collecting into ACSF containing 1% FBS. Cell viability exceeded 80% for all samples. Sorted cells were collected by centrifugation and resuspended in 1× DPBS containing 0.04% BSA. Single-cell RNA sequencing libraries were prepared using the Chromium Single Cell 3′ GEM, Library & Gel Bead Kit v3 (10× Genomics, Pleasanton, CA, USA). Samples were loaded onto the Chromium Controller (10× Genomics, Pleasanton, CA, USA) to generate gel bead-in-emulsions (GEMs), with target cell recovery varying by species (1000–9000 cells per replicate depending on sample availability). Reverse transcription was performed using a C1000 Touch Thermal Cycler (Bio-Rad Laboratories, Hercules, CA, USA), and subsequent cDNA amplification and library construction were carried out according to the manufacturer’s instructions (10× Genomics). Libraries were sequenced on an Illumina HiSeq X platform (paired-end 100 bp reads) targeting an average of 20,000 read pairs per cell.

### 4.5. scRNA-seq Data Processing

Raw FASTQ files were processed using Cell Ranger (v7.2.0). Reference genomes and gene annotations for mudskipper (*P. magnuspinnatus*) and chicken (*G. gallus*) were obtained from Ensembl (release 113) [42]. Because a reference genome for the fugu species used in this study (*T. obscurus* X *T. rubripes*) was not available, fugu reads were mapped to a custom reference built from the genome assembly of the closely related congeneric species *T. rubripes* [43,44], retrieved from Ensembl (release 113) [42]. For all species, custom Cell Ranger-compatible references were generated from the respective genome assemblies and gene annotations. Mapping statistics for all newly generated datasets are summarized in Table S7.

### 4.6. scRNA-seq Quality Control

Raw gene–barcode matrices generated from the 10× Genomics platform were imported into R using DropletUtils (v1.26.0) and processed as SingleCellExperiment (v1.28.0) objects [51]. Empty droplets were removed using emptyDrops, retaining barcodes with FDR ≤ 0.01. Per-cell quality control metrics were calculated using addPerCellQC in scater (v1.34.1) [52]. Because each dataset comprised two replicates, sample-specific cutoffs were applied according to the distribution of QC metrics in each sample. For fugu, cells with >3000 UMIs and >90 detected genes (replicate C4) or >3000 UMIs and >80 detected genes (replicate C5) were retained. For mudskipper, cells with >1600 UMIs and >75 detected genes (replicate C6) or >1700 UMIs and >70 detected genes (replicate C7) were retained. For chicken, cells with >5000 UMIs and >150 detected genes (replicate Ch1) or >5500 UMIs and >160 detected genes (replicate Ch2) were retained. Doublets were identified using scDblFinder (v1.23.4), and only singlets were included in downstream analyses [53].

For the mouse dataset [21], which was generated using 10× snRNA-seq, raw count matrices were extracted from the published Seurat RDS objects, and cell type annotations were assigned based on the cluster identities stored within the original Seurat objects. Only uninjured control samples were retained to ensure comparison of homeostatic transcriptional states. The extracted count data were subsequently reprocessed through our unified analysis pipeline for normalization and clustering to ensure consistency with the in-house datasets. For the human dataset [22], generated using a combined 10× scRNA-seq and snRNA-seq approach, quality-controlled count matrices and associated metadata were obtained from the Gene Expression Omnibus (GEO; accession GSE188516). The pre-filtered data were imported using read10xCounts in DropletUtils (v1.26.0), and cell type annotations were assigned based on the metadata provided by the original authors. Both public datasets were subsequently reprocessed through our unified analysis pipeline for normalization and clustering to ensure consistency with the in-house datasets. Comprehensive technical metrics for all datasets used in this study are summarized in Table S9.

### 4.7. scRNA-seq Data Analysis

After quality control, filtered datasets were analyzed in Seurat (v5.3.0) [23]. Data were processed using NormalizeData, FindVariableFeatures, ScaleData, and RunPCA, followed by graph-based clustering with FindNeighbors and FindClusters and visualization by RunUMAP. Cell identities were assigned on the basis of canonical marker genes and cluster-enriched markers identified using FindAllMarkers and FindMarkers.

For oligodendrocyte lineage analysis, oligodendrocyte populations from each species were subsetted from the spinal cord datasets and analyzed using Monocle3 (v1.3.1) following conversion from Seurat objects with SeuratWrappers (v0.4.0) [26]. Trajectory roots were determined using an OPC root score calculated from the combined expression of progenitor markers (*Pdgfra*, *Nkx2-2*, *Sox2*, *Cspg5*, *Tnr*). The top 10% of cells by root score were used to identify the principal graph node closest to the OPC state, which was set as the trajectory root. Cells were then ordered in pseudotime along the inferred trajectory.

Stage boundaries (OPC/COP and COP/MOL) were defined using module score crossover points. Stage-specific marker gene sets for OPC, COP, and MOL were curated from established oligodendrocyte lineage studies in mouse and human [21,22]. The OPC module included *Pdgfra*, *Nkx2-2*, *Sox2*, *Cspg5*, and *Tnr*; the COP module included *Fyn* and *Tcf7l2*; and the MOL module included *Plp1*, *Mag*, *Mbp*, and *Mog*. Orthologous genes were identified in each species using one-to-one ortholog mappings derived from OrthoFinder. Module scores for each stage were calculated using Seurat’s AddModuleScore function, and boundaries were set at pseudotime values where module score crossover occurred: the OPC/COP boundary was set where the COP module score exceeded the OPC module score, and the COP/MOL boundary where the MOL module score surpassed the COP module score.

### 4.8. CAME-Based Cross-Species Cell Type Mapping

Cross-species cell type mapping was performed using CAME (v0.1.13) in Python (v3.8.20) with Scanpy (v1.9.8) [29,54]. Analyses were conducted in unaligned mode using a mouse reference–query framework. The mouse oligodendrocyte lineage dataset (OPC, COP, and MOL) was used as the reference, and oligodendrocyte lineage cells from fugu, mudskipper, chicken, and human were used as query datasets. Mouse-to-species ortholog mapping tables, including both one-to-many and one-to-one ortholog relationships, were used as input. Genes detected in fewer than three cells were removed from each dataset. CAME preprocessing was performed using preprocess_unaligned, and model training and prediction were performed using main_for_unaligned with use_scnets=True, keep_non1v1_feats=True, ntop_deg_nodes=50, ntop_deg=200, and n_epochs=400. Prediction probability score matrices were extracted and used for downstream visualization. To assess whether differences in COP mapping between amniote and teleost species were attributable to cell number, we performed downsampling analysis on the chicken and human datasets. Random downsampling was performed by randomly selecting 2300 cells (matching the smallest dataset, fugu) without regard to cell type composition. CAME was re-run on each downsampled dataset using the same parameters described above.

### 4.9. Identification of Dynamically Expressed Genes and Conserved Gene Modules

For each species, dynamically expressed genes along the oligodendrocyte differentiation trajectory were identified using stage-based DEG analysis. Using the ortholog-converted expression matrices, Seurat’s FindMarkers function was applied with a Wilcoxon rank-sum test for each differentiation stage (OPC, COP, and MOL) against all other stages, restricted to genes present in the common ortholog set across all five species. Genes with an adjusted *p*-value < 0.05 and log_2_ fold change > 0.25 were considered significantly dynamic. For each species, dynamic genes from all three stages were combined into a single list per species. Gene identities were harmonized across species using one-to-one ortholog mappings to mouse gene symbols (Table S2). Genes identified as dynamically expressed in at least four of the five species (≥80%) were defined as high-shared genes, resulting in 302 core conserved genes (Table S3).

For module identification, pseudotime values were normalized to a 0–1 scale within each species to enable cross-species comparison. Pseudobulk expression profiles were generated by binning cells into 10 equally spaced pseudotime intervals per species and computing the mean normalized expression per bin. Expression values were z-score normalized per gene across all bins and species. Hierarchical clustering (ward.D2 method, Euclidean distance) was applied to the concatenated pseudobulk matrix to identify co-expression modules, and the dendrogram was cut into four modules (k = 4) based on visual inspection of module coherence and temporal expression patterns. Gene Ontology biological process enrichment analysis was performed on each module using enrichGO in clusterProfiler (v4.14.0) with the org.Mm.eg.db (v3.20.0) annotation database (Benjamini–Hochberg adjusted *p*-value < 0.05, *q*-value < 0.1). Redundant GO terms were reduced using the simplify function with a semantic similarity cutoff of 0.8, and pairwise term similarity was computed using pairwise_termsim for enrichment map network visualization via emapplot in the enrichplot (v1.26.1) package, displaying up to 10 terms per module. The full GO enrichment results for each conserved gene module are provided in Table S6. Module score dynamics along normalized pseudotime were calculated as the mean z-score of all genes within each module per pseudotime bin. Representative gene expression dynamics were computed by dividing normalized pseudotime into 20 bins and calculating mean expression per bin, with LOESS curves (span = 0.6) fitted for each species.

### 4.10. Stage-Specific DEG and TF Analysis

To identify stage-specific differentially expressed genes (DEGs) for cross-species comparison (Figure S5), pairwise comparisons between each oligodendrocyte stage (OPC, COP, and MOL) and all other stages were performed within each species using Seurat’s FindMarkers function with a Wilcoxon rank-sum test (adjusted *p*-value < 0.05, log_2_ fold change > 0.25, minimum expression fraction > 0.1). Gene identifiers were harmonized to mouse gene symbols using one-to-one ortholog mappings. DEGs were classified by conservation level based on the number of species in which each gene was identified as differentially expressed in the same stage, with genes shared by ≥4 species classified as conserved and genes detected in only one species classified as species-specific (Table S4). For each species, pseudobulk expression values were calculated per stage, z-score normalized per gene, and visualized as heatmaps using ComplexHeatmap (v2.22.0).

For TF analysis (Figure S6), a curated list of mouse TFs was obtained from Gene Ontology term GO:0003700 (DNA-binding transcription factor activity) via the org.Mm.eg.db (v3.20.0) annotation database. TFs were identified from the stage-specific DEG lists (adjusted *p*-value < 0.05, log_2_ fold change > 0.25). Conservation was assessed on a same-stage basis: for each stage, the number of species in which each TF was identified as significantly enriched was determined. TFs detected in ≥4 species within the same stage were classified as shared, and TFs detected in only one species were classified as species-specific (Table S5). For visualization, the top five species-specific TFs per species were selected based on log_2_ fold change ranking. Pseudobulk expression values per stage were calculated from ortholog-converted expression matrices, z-score normalized, and displayed as heatmaps with conservation-level annotations using ComplexHeatmap (v2.22.0).

### 4.11. Aquatic–Terrestrial Divergence Analysis

To examine the divergence of oligodendrocyte differentiation programs along the aquatic–terrestrial axis, stage-specific DEGs were compared between amniote (chicken, mouse, and human) and teleost (fugu and mudskipper) species. Amniote-exclusive genes were defined as DEGs shared among all three amniote species but absent from both teleost species. Teleost-exclusive genes were defined as DEGs shared between both teleost species but absent from all three amniote species. Evolutionary core genes were defined as DEGs detected in all five species. For each gene group, stage enrichment was determined by assigning each gene to the stage in which it was most frequently detected as a DEG across the contributing species. GO biological process enrichment analysis was performed on the amniote-exclusive and evolutionary core gene sets using enrichGO in clusterProfiler (v4.14.0) with the org.Mm.eg.db (v3.20.0) annotation database. For representative gene visualization, pseudotime was normalized to a 0–1 scale within each species, binned into 20 intervals, and mean expression per bin was calculated. LOESS curves (span = 0.6) were fitted for each species.

### 4.12. Postnatal Mouse Validation Analysis

To assess the impact of developmental stage on cross-species DEG comparisons, we analyzed postnatal mouse spinal cord data from Rosenberg et al. (2018) (GSE110823) [24], generated using SPLiT-seq. The published .mat file (GSM3017261_150000_CNS_nuclei.mat) containing 156,049 nuclei was loaded in Python (v3.12.10) using scipy.io, and spinal cord cells (P2 and P11; 22,614 nuclei) were extracted based on the sample_type annotation. Cells annotated as “Unresolved”, “Unassigned”, or “NA” in the spinal_cluster_assignment field were excluded, yielding 14,697 cells for analysis. Cell types were assigned based on the original spinal cluster annotations: oligodendrocyte lineage cells (OPC, Committed Oligodendrocyte Precursor Cells, Oligo Mature, Oligodendrocyte Myelinating, Oligo COP1, Oligo COP2, Oligo NFOL1, Oligo MFOL1, Oligo MFOL2, Oligo MOL) were classified as “Oligodendrocyte”, while excitatory neurons, inhibitory neurons, and motor neurons were classified as “Neuron”, and remaining cell types were annotated accordingly. The count matrix and metadata were exported in Matrix Market format for downstream analysis in R (v4.4.3).

The spinal cord dataset was processed in Seurat (v5.3.0) using the same pipeline as the other species: NormalizeData, FindVariableFeatures, ScaleData, RunPCA (10 PCs), FindNeighbors, FindClusters (resolution 0.5), and RunUMAP. Oligodendrocyte lineage cells (3973 cells) were subset and reprocessed independently using RunPCA (4 PCs), FindNeighbors, FindClusters (resolution 0.3), and RunUMAP. Pseudotime trajectory analysis was performed using Monocle3 with the same root selection strategy (OPC markers: *Pdgfra*, *Nkx2-2*, *Cspg5*, *Tnr*) and module score-based stage annotation (OPC, COP, and MOL) as described above. The cross-species DEG analysis was then repeated with the postnatal mouse dataset replacing the adult mouse dataset, while retaining the same data for the other four species, using identical parameters for ortholog conversion, FindMarkers, and conservation-level classification.

### 4.13. Graphics and Statistical Analysis

All figures were composed and adjusted using Adobe Illustrator (v30.4; Adobe Inc., San Jose, CA, USA). Statistical analyses and visualizations were performed in R (v4.4.3) using ggplot2 (v3.5.2), ComplexHeatmap (2.22.0), pheatmap (v1.0.13), and UpSetR (v1.4.0). DEG analyses employed the Wilcoxon rank-sum test as implemented in Seurat’s FindMarkers function, with Bonferroni correction for multiple testing.

## Figures and Tables

**Table 1:** Summary of samples used in this study.

Species	Stage	*n* (Replicates)	Total Cells (After QC)	Platform	Source
Fugu	Juvenile (~1 month post hatching)	2	4771	scRNA-seq (10× Chromium 3′ v3)	GSE328630
Mudskipper	Juvenile (~1 month post hatching)	2	5548	scRNA-seq (10× Chromium 3′ v3)	GSE328630
Chicken	HH36-37	2	14,816	scRNA-seq (10× Chromium 3′ v3)	GSE328630
Mouse (adult) [21]	Adult (12–30 week)	3	10,633	10× snRNA-seq (10× Chromium 3′ v2)	GSE172167
Human [22]	GW17-18	4	147,438	scRNA + snRNA-seq (10× Chromium 3′ v3)	GSE188516
Mouse (postnatal) [24]	Postnatal day 2 and 11	2	14,697	scRNA-seq (SPLiT-seq)	GSE110823

## Data Availability

The raw data supporting the conclusions of this article will be made available by the authors on request.

## Supplementary Materials

The following supporting information can be downloaded at: https://www.mdpi.com/article/10.3390/ijms27104283/s1.
