# Supplementary material for: Shared and Divergent Transcriptional Programs of Oligodendrocyte Differentiation Across Vertebrate Species Revealed by scRNA-seq Analysis
Source: Int J Mol Sci. 2026 May 11;27(10):4283. doi: 10.3390/ijms27104283 (PMC13208018; doi:10.3390/ijms27104283)
Supplement: Supplementary file 1 [file ijms-27-04283-s001.zip › Oligo-Supplementary-Materials-Final.pdf]

Supplementary Materials for

Shared and divergent transcriptional programs of oligodendrocyte differentiation across vertebrate species revealed by scRNA-seq analysis

Tery Yun, Junhee Park, Myungin Baek*

Department of Brain Sciences, DGIST, Daegu 42988, Republic of Korea
*Correspondence: bmi008@dgist.ac.kr (M.B.)

This PDF file includes:

Supplementary Fig. S1 to S13

Supplementary Figure S1.

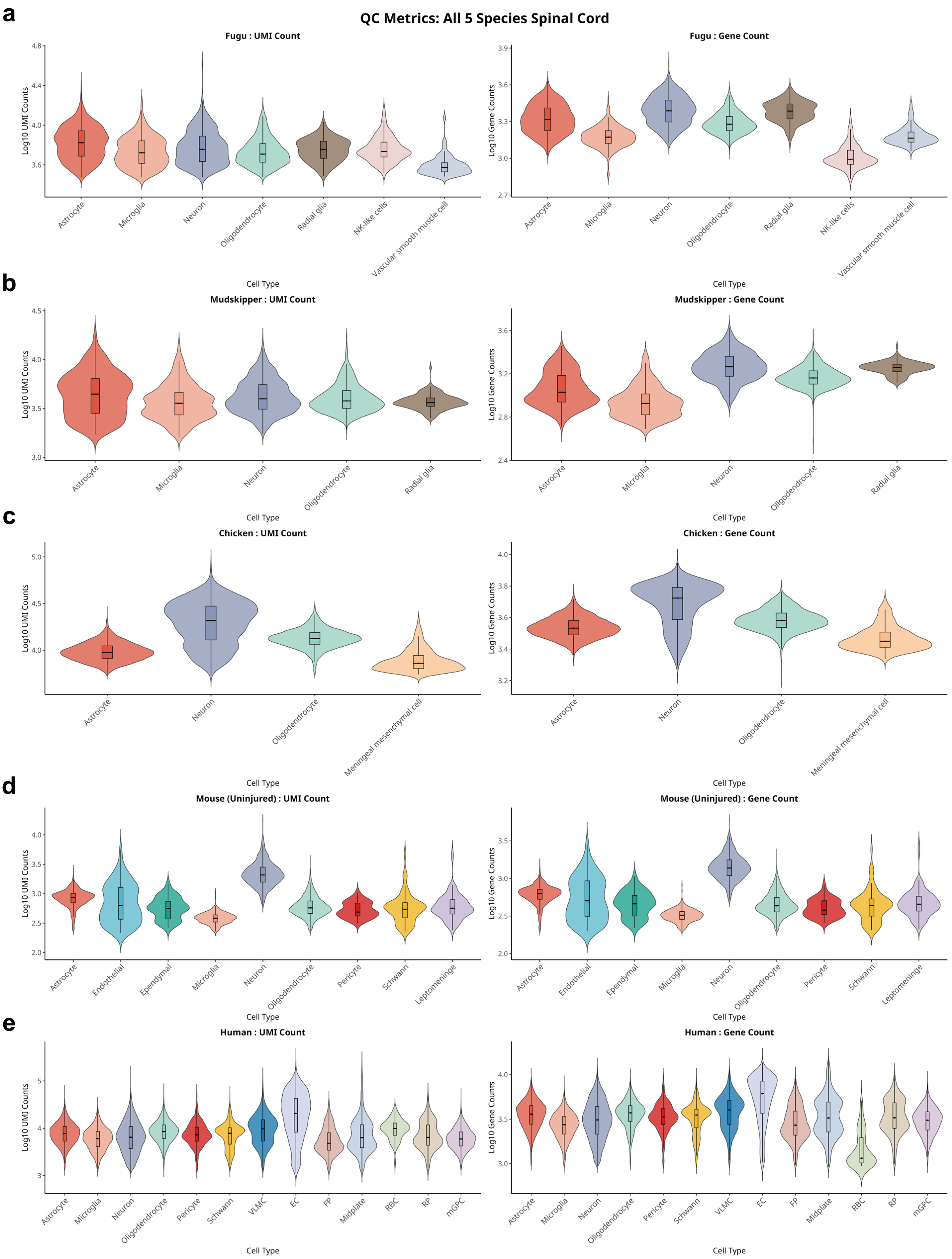

Supplementary Figure S1. Quality control metrics for spinal cord scRNA-seq datasets. (**a**–**e**) Violin plots showing the distribution of log10-transformed UMI counts (left) and detected gene counts (right) per cell for each cell type in **(a)** fugu, **(b)** mudskipper, **(c)** chicken, **(d)** mouse, and **(e)** human spinal cord datasets. Boxplots within violins indicate the median and interquartile range.

Supplementary Figure S2.

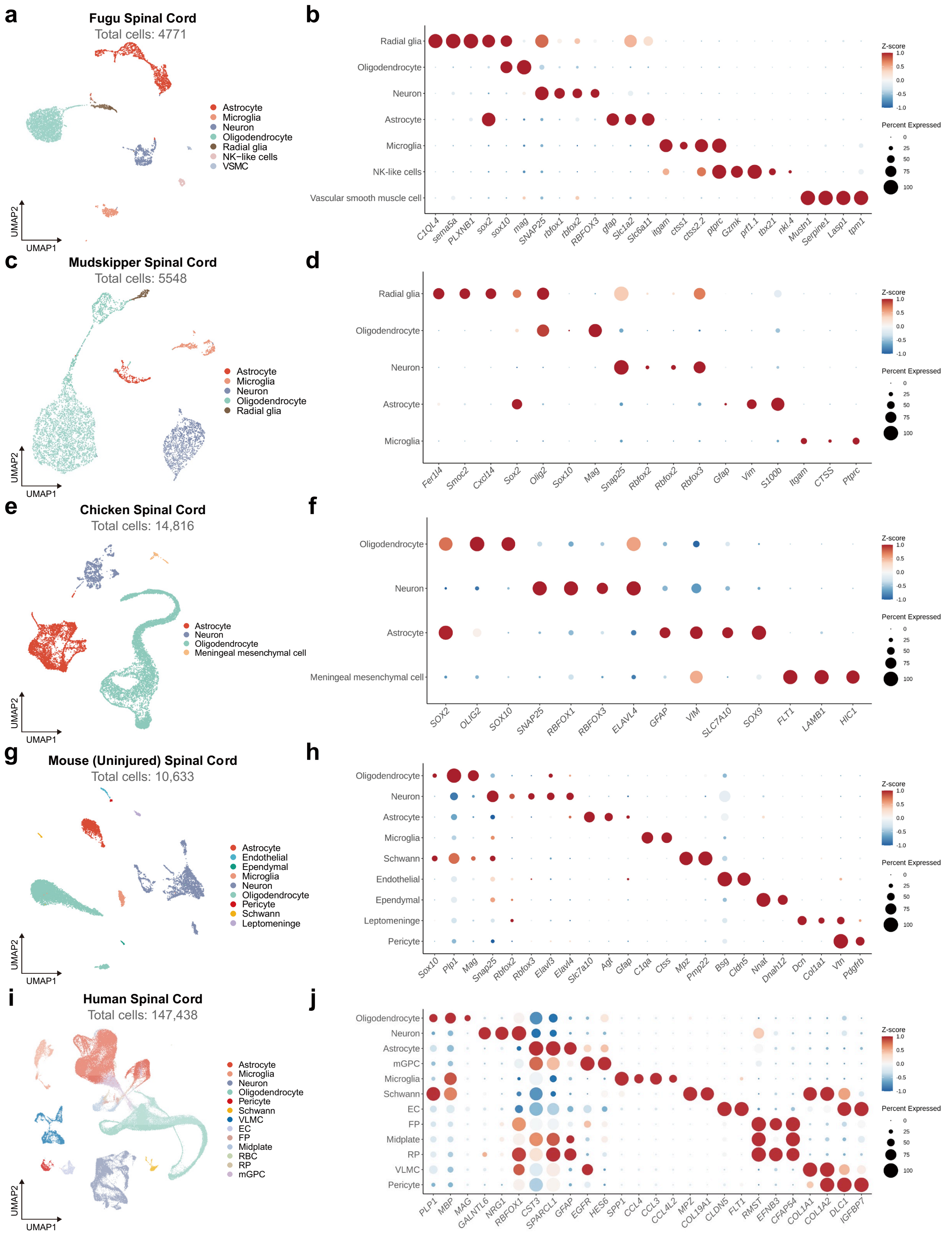

Supplementary Figure S2. Cell type annotation of five vertebrate spinal cords.

(**a**–**j**) UMAP visualizations and dot plots of canonical marker gene expression for **(a,b)** fugu, **(c,d)** mudskipper, **(e,f)** chicken, **(g,h)** mouse, and **(i,j)** human spinal cord datasets. Dot size represents the percentage of cells expressing each marker gene; dot color indicates the Z-score-normalized mean expression level.

Supplementary Figure S3.

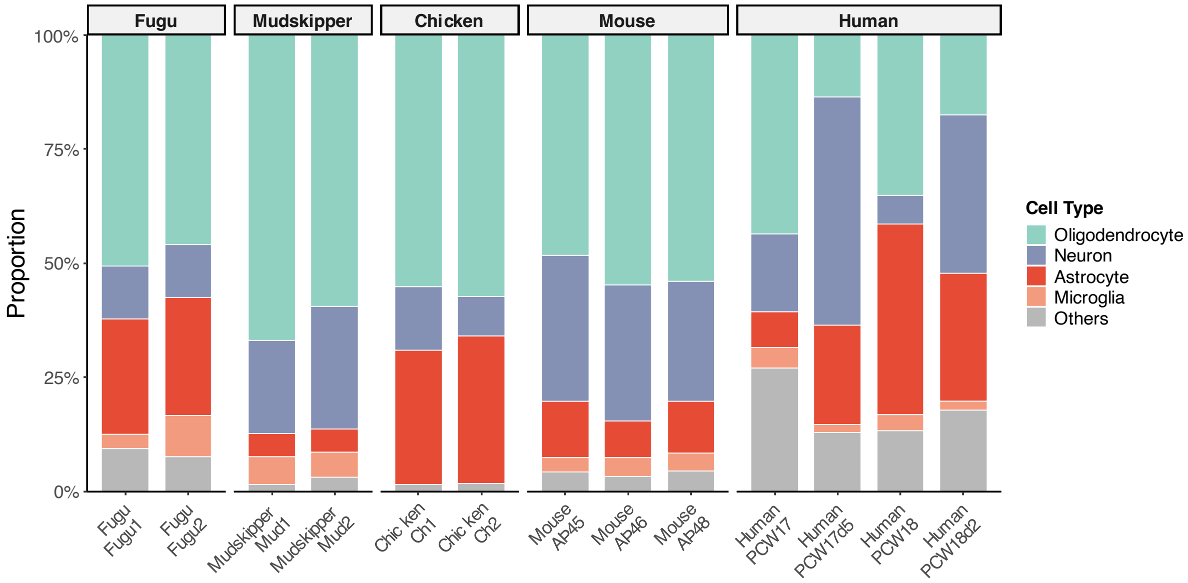

Supplementary Figure S3. Cell type proportions across biological replicates within each species.

Stacked bar plots showing the proportion of oligodendrocytes, neurons, astrocytes, microglia, and other cell types for each biological replicate. Fugu (Fugu1, Fugu2; N=2), mudskipper (Mud1, Mud2; N=2), and chicken (Ch1, Ch2; N=2) represent newly generated datasets in this study. Mouse replicates (A-45, A-46, A-48; N=3) were derived from uninjured adult spinal cord samples from Matson et al. (2022) (GSE172167). Human samples (PCW17, PCW17d5, PCW18, PCW18d2; N=4) were derived from gestational week 17–18 spinal cord tissue from Andersen et al. (2023) (GSE188516), comprising both single-cell and single-nucleus preparations. Cell type proportions were consistent between biological replicates within each species, supporting the reliability of the cross-species comparisons presented in Figure 1h.

Supplementary Figure S4.

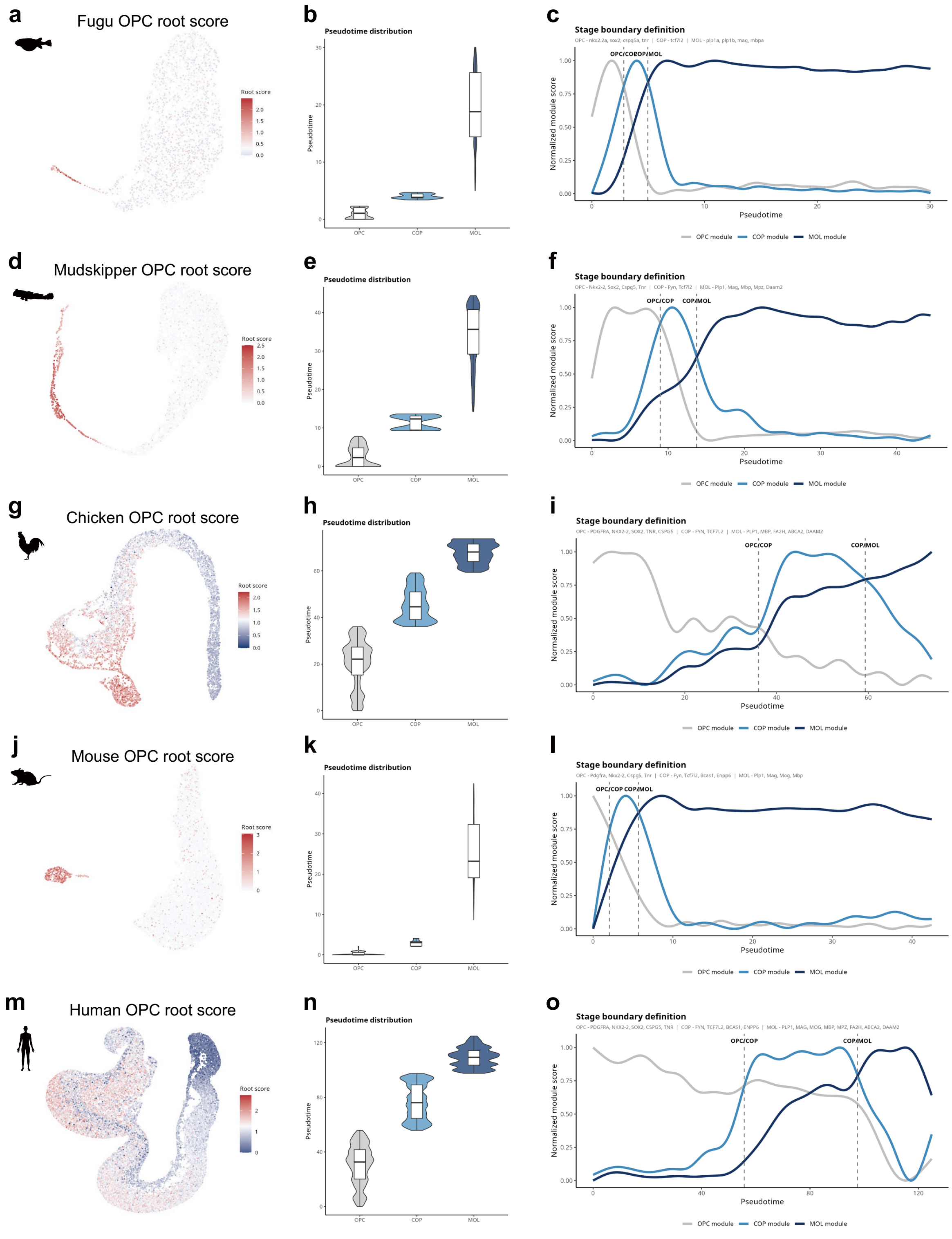

Supplementary Figure S4. Pseudotime trajectory construction and stage boundary definition.

(**a**–**o**) For each species (fugu, **a**–**c**; mudskipper, **d**–**f**; chicken, **g**–**i**; mouse, **j**–**l**; human, **m**–**o**), shown on left: UMAP colored by OPC root score; middle: Violin plots of pseudotime distribution per stage, and right: normalized module score dynamics along pseudotime, with dashed lines indicating stage boundaries.

Supplementary Figure S5.

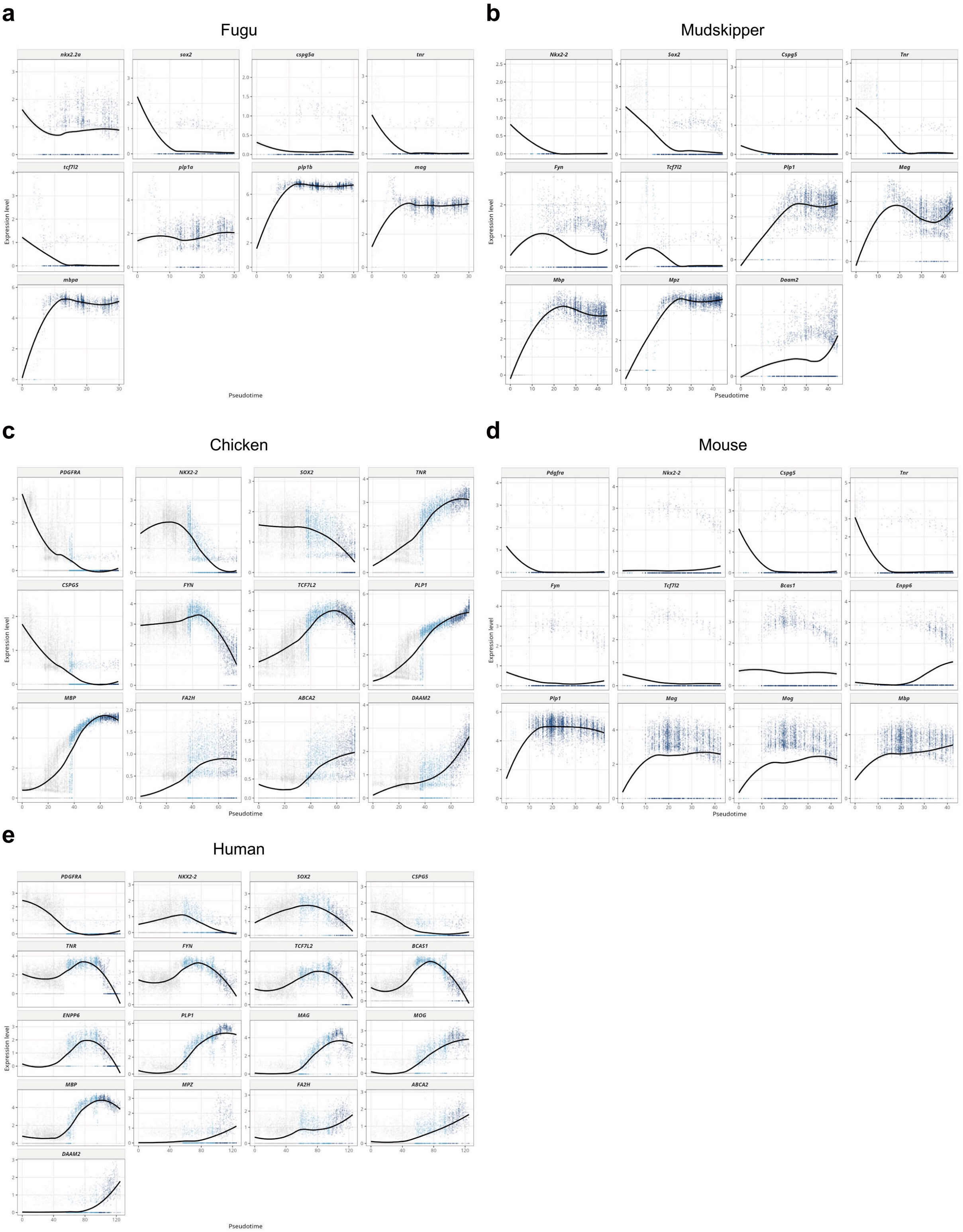

Supplementary Figure S5. Individual gene expression dynamics along pseudotime. (**a**–**e**) Scatter plots with LOESS-fitted curves showing representative marker gene expression as a function of pseudotime for **(a)** fugu, **(b)** mudskipper, **(c)** chicken, **(d)** mouse, and **(e)** human.

Supplementary Figure S6.

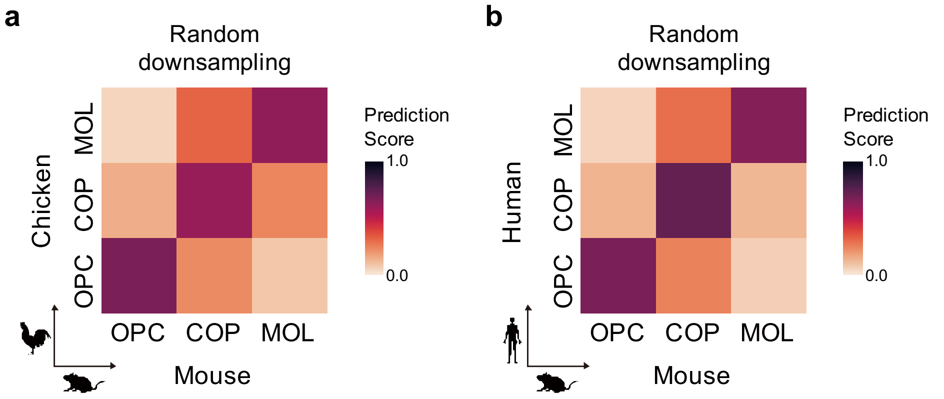

Supplementary Figure S6. CAME cross-species cell type mapping after random downsampling of chicken and human datasets.

Prediction score heatmaps from CAME analysis using mouse as the reference species. Chicken **(a)** and human **(b)** datasets were randomly downsampled to 2,300 cells to match fugu, which had the fewest cells among the five species. Both species preserved robust diagonal mapping patterns, including clear COP-to-COP mapping, indicating that total cell number does not account for the reduced COP mapping observed in teleosts (Figure 3a). Color scale represents the mean prediction score for each cell type pair.

Supplementary Figure S7.

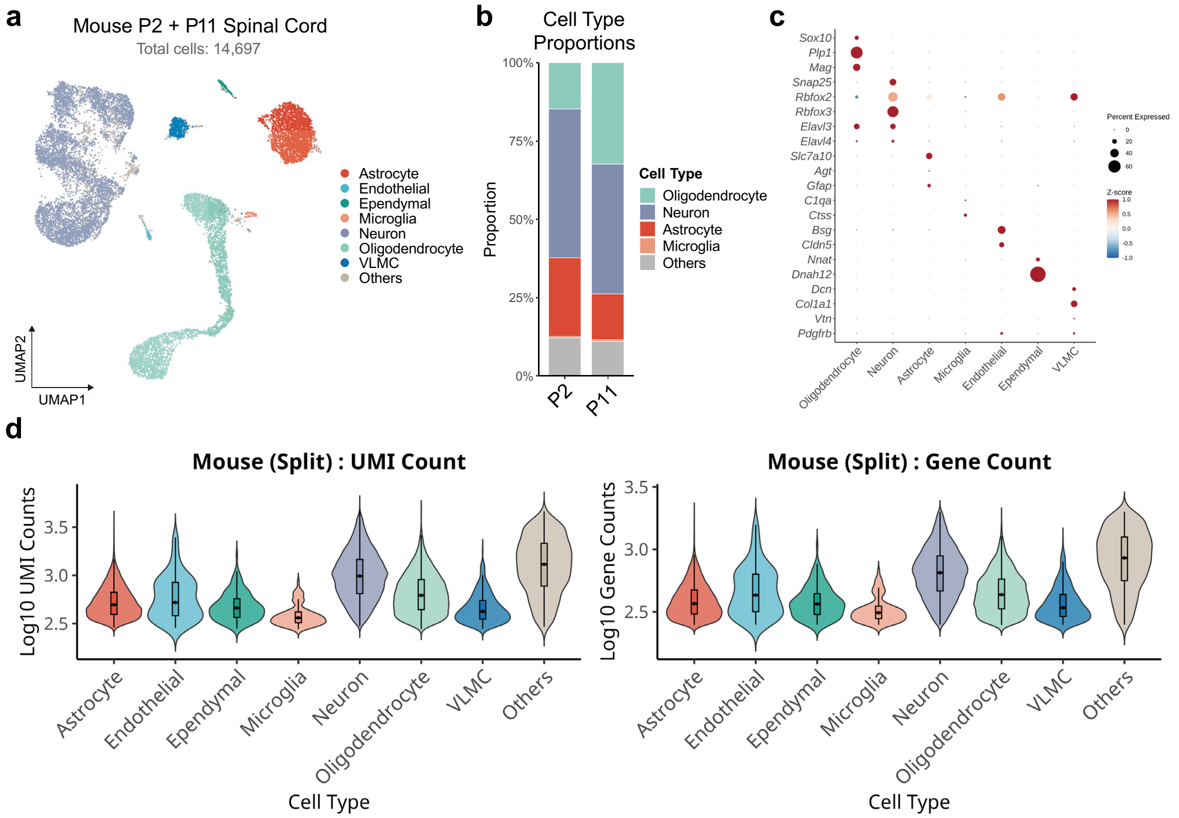

Supplementary Figure S7. Postnatal mouse spinal cord overview (SPLiT-seq, P2+P11). (**a**) UMAP visualization of postnatal mouse spinal cord cells (P2 and P11 combined; 14,697 cells after removal of unresolved clusters) colored by cell type. (**b**) Stacked bar plot showing cell type proportions for P2 and P11 samples. (**c**) Dot plot of canonical cell type marker gene expression across annotated cell types. Dot size represents the percentage of cells expressing each gene; color intensity represents the average scaled expression (Z-score). (**d**) Violin plots of UMI counts per cell (left) and genes detected per cell (right) across cell types. Data were generated by SPLiT-seq (Rosenberg et al., 2018; GSE110823) from P2 and P11 mouse spinal cord.

Supplementary Figure S8.

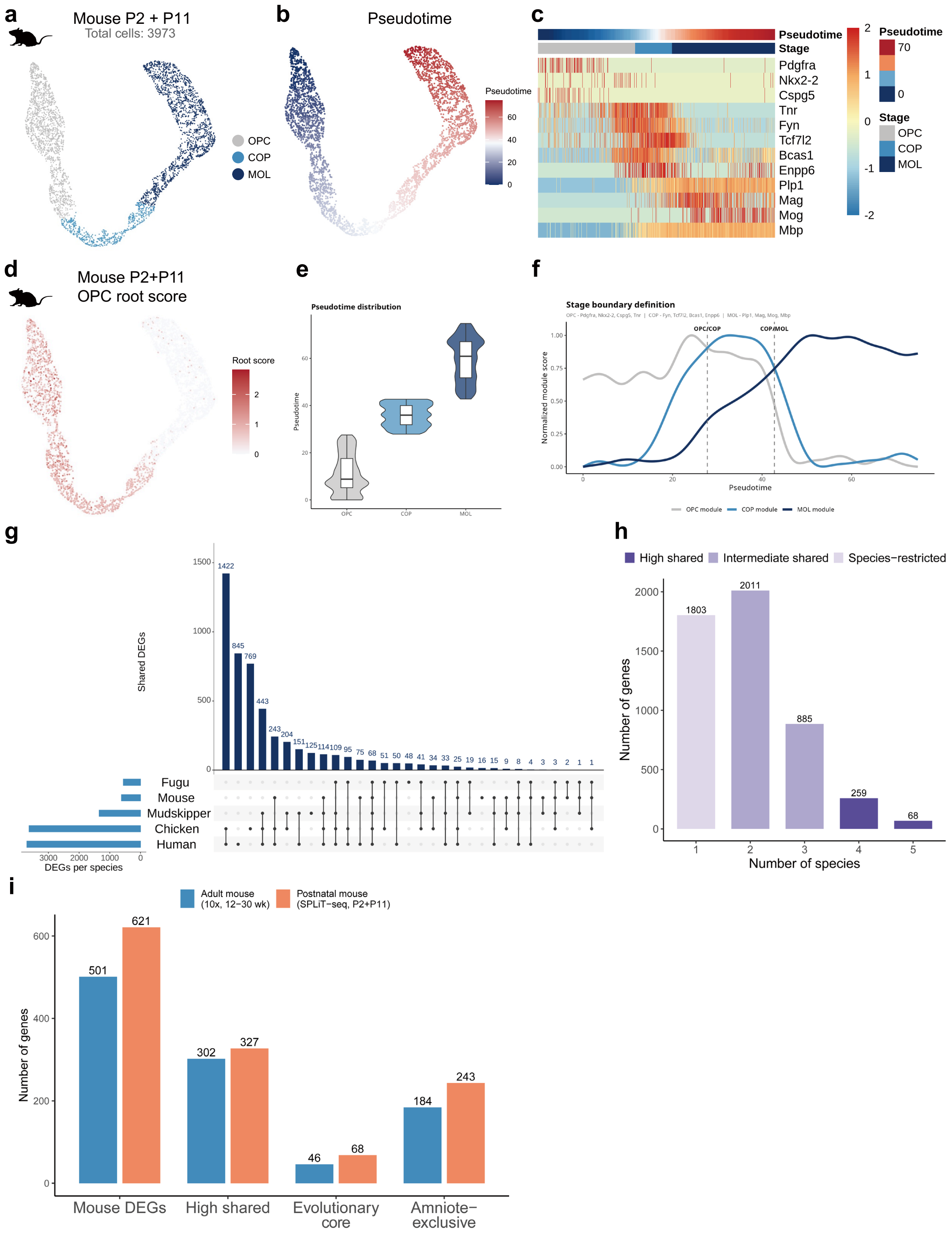

Supplementary Figure S8. Postnatal mouse oligodendrocyte pseudotime analysis and cross-species comparison.

(**a**) UMAP of oligodendrocyte lineage cells (3,973 cells) colored by stage (OPC, COP, MOL), showing a continuous differentiation trajectory in contrast to the spatial separation observed in the adult mouse dataset (Figure 2j). (**b**) Pseudotime UMAP confirming a continuous OPC-to-MOL gradient. (**c**) Heatmap of marker gene expression ordered by pseudotime, with OPC markers (*Pdgfra*, *Nkx2-2*, *Cspg5*, *Tnr*) at low pseudotime and MOL markers (*Plp1*, *Mag*, *Mog*, *Mbp*) at high pseudotime. (**d**) OPC root score distribution on UMAP, confirming correct pseudotime root assignment. (**e**) Violin plot of pseudotime distribution across OPC, COP, and MOL stages. (**f**) Stage boundary definition using normalized module scores for OPC, COP, and MOL marker gene sets. (**g**) UpSet plot showing the intersection of stage-specific DEGs across five species when the postnatal mouse dataset replaces the adult mouse dataset. (**h**) Gene sharing distribution showing 327 high shared genes (≥4 species) including 68 evolutionary core genes (all 5 species). (**i**) Grouped bar plot comparing four key cross-species metrics between the adult mouse dataset (10x Chromium, 12–30 weeks) and the postnatal mouse dataset (SPLiT-seq, P2+P11). DEGs were identified using genes with one-to-one orthologs shared across all five species. All metrics increased with the postnatal dataset: mouse DEGs (501 to 621), high shared genes (302 to 327), evolutionary core genes (46 to 68), and amniote-exclusive genes (184 to 243), confirming that the main analysis using adult mouse data provides a conservative estimate of cross-species gene sharing.

Supplementary Figure S9.

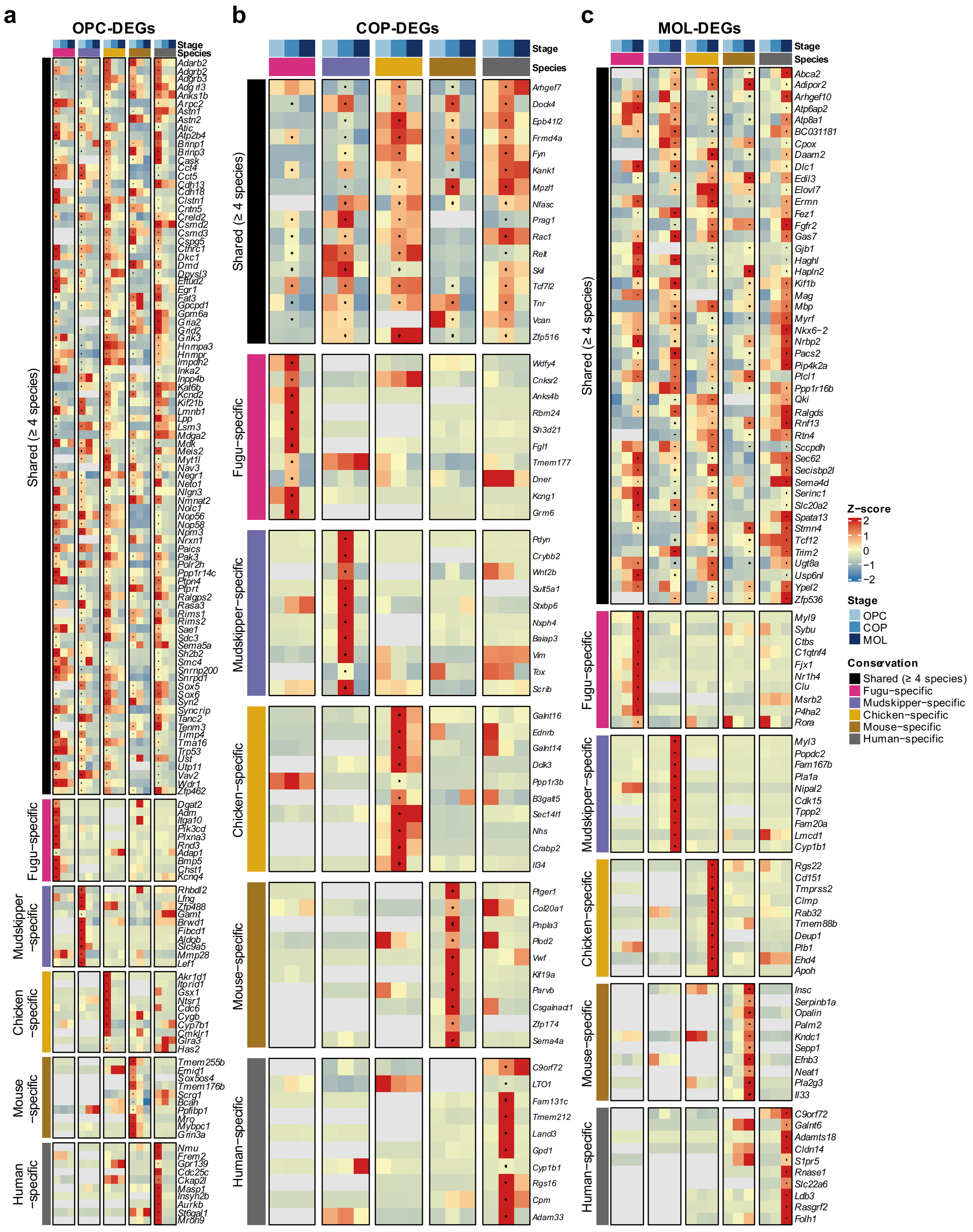

Supplementary Figure S9. Stage-specific DEGs across five species.

(**a**–**c**) Heatmaps of stage-specific DEGs for **(a)** OPC, **(b)** COP, and **(c)** MOL. For each stage, genes are grouped by conservation level: shared across ≥4 species (top, black side bar) and species-specific (bottom, colored side bars indicating individual species). Columns represent pseudotime-binned expression (OPC, COP, MOL stages) for each of the five species, with expression values shown as Z-scores. Black dots indicate species in which the gene was identified as significantly differentially expressed (Wilcoxon rank-sum test, adjusted p-value < 0.05, $\log_2$ fold change > 0.25). Gene names are displayed as mouse ortholog symbols.

Supplementary Figure S10.

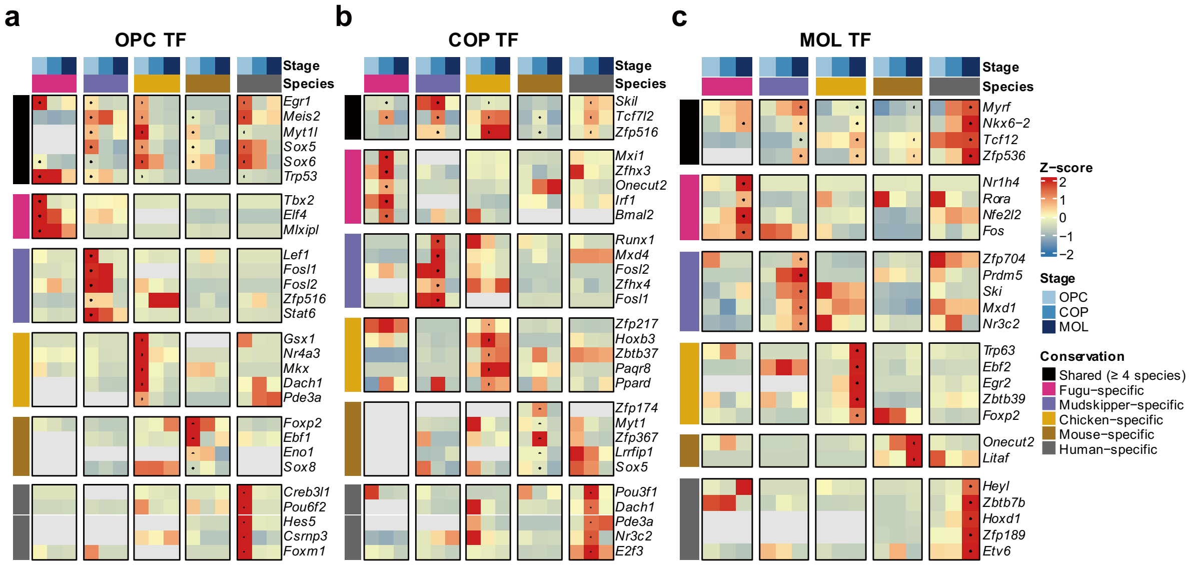

Supplementary Figure S10. Stage-specific TFs across five species.
Heatmaps of TFs expression for **(a)** OPC, **(b)** COP, and **(c)** MOL stages. TFs are grouped by conservation level: shared across ≥4 species (top, black side bar) and species-specific (bottom, colored side bars). Expression values are shown as Z-scores across pseudotime-binned OPC, COP, and MOL stages for each species. Black dots indicate species in which the TF was identified as significantly enriched (adjusted p-value < 0.05, $\log_2$ fold change > 0.25).

Supplementary Figure S11.

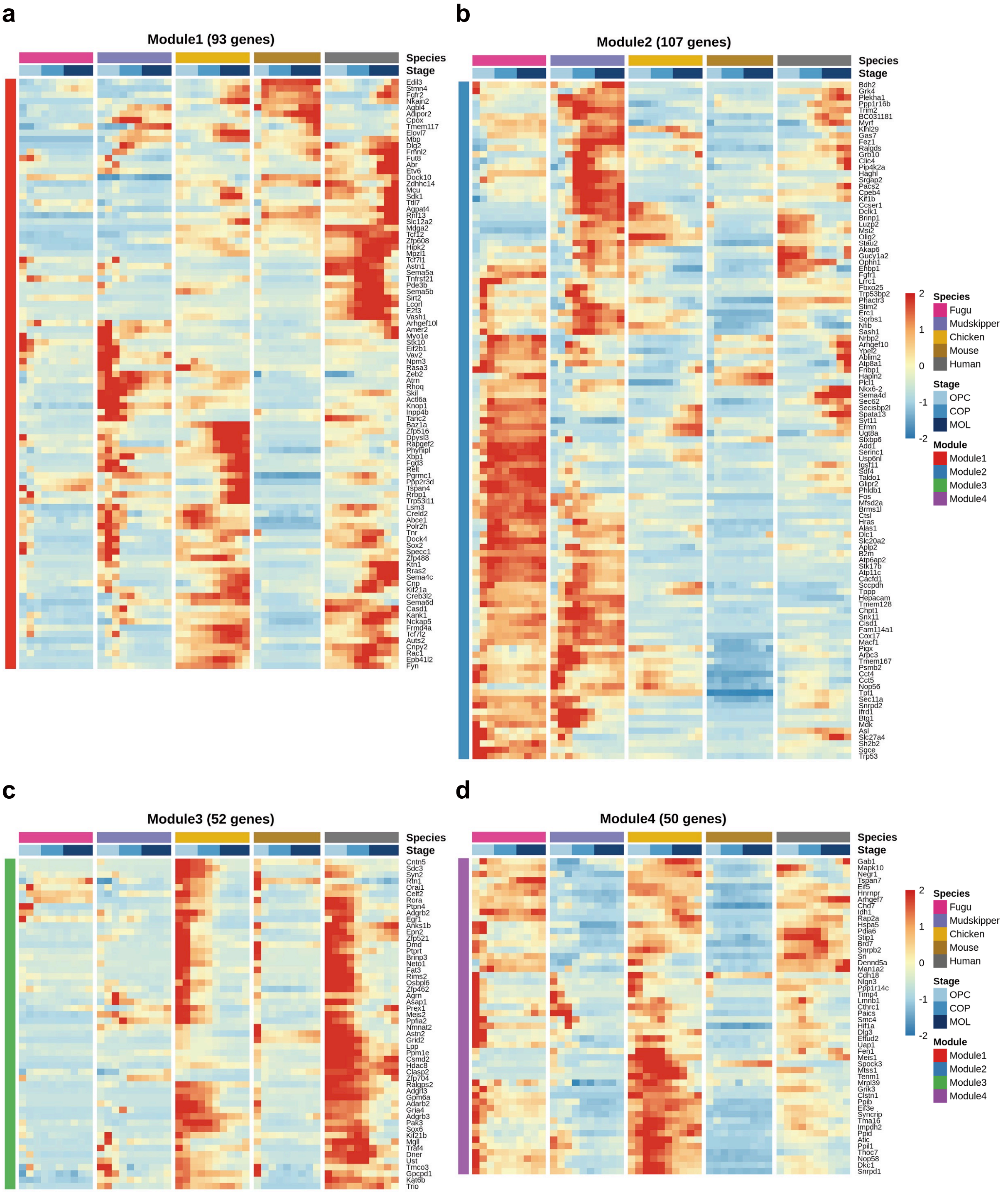

Supplementary Figure S11. Expression heatmaps of conserved gene modules. (**a**–**c**) Heatmaps of all genes within **(a)** Module 1 (93 genes), **(b)** Module 2 (107 genes), **(c)** Module 3 (52 genes), and **(d)** Module 4 (50 genes) across five species, shown as pseudotime-binned Z-scores for OPC, COP, and MOL stages.

Supplementary Figure S12.

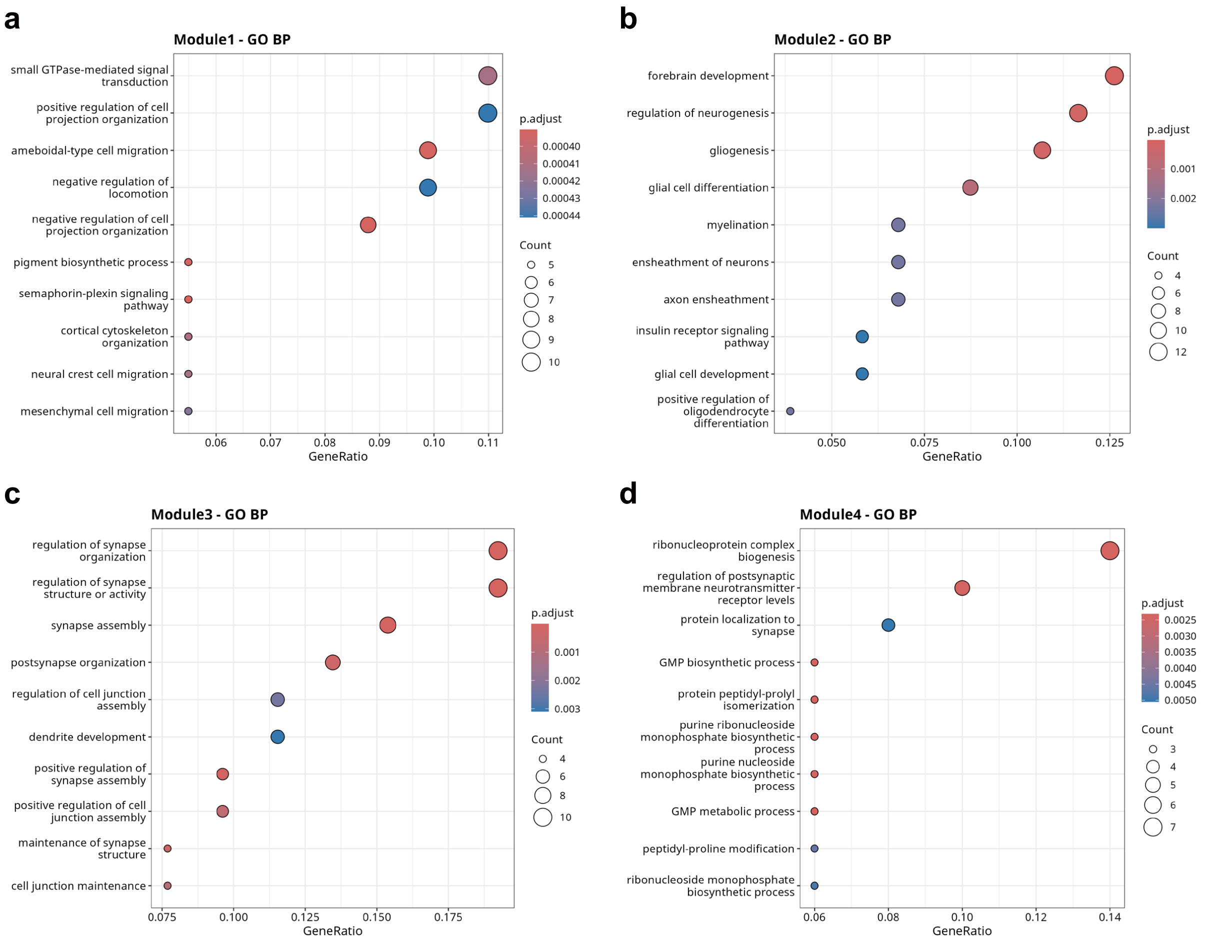

Supplementary Figure S12. GO biological process enrichment for conserved gene modules.
(**a**–**c**) Dot plots showing GO BP enrichment for **(a)** Module 1, **(b)** Module 2, **(c)** Module 3, and **(d)** Module 4. Dot size represents gene count; dot color represents adjusted *p*-value.

Supplementary Figure S13.

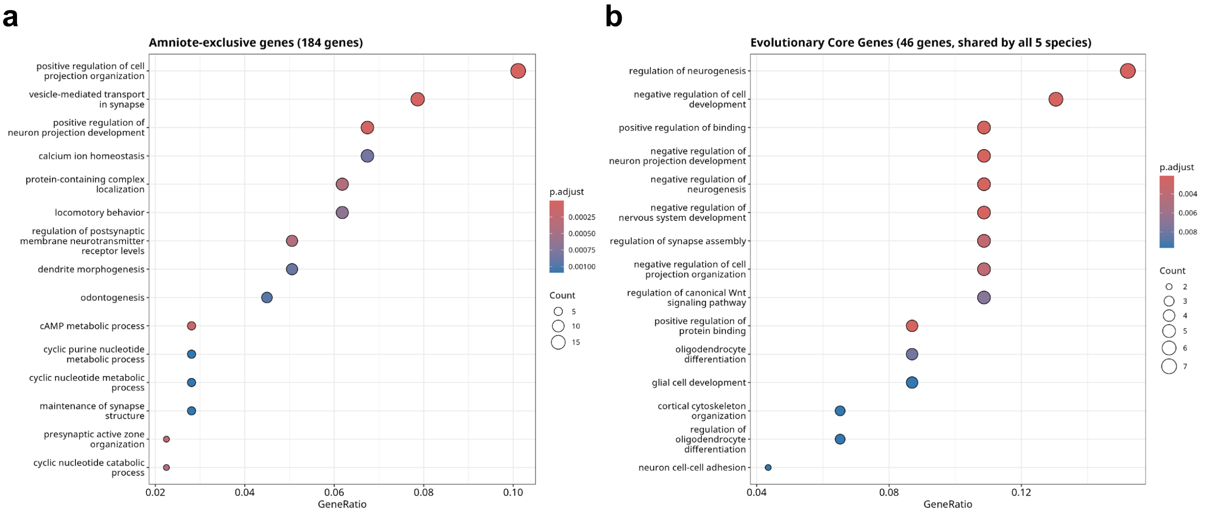

Supplementary Figure S13. GO biological process enrichment analysis of amniote-exclusive and evolutionary core genes.

(a) GO dotplot for amniote-exclusive genes (184 genes shared by chicken, mouse, and human but absent in both teleosts).

(b) GO dotplot for evolutionary core genes (46 genes shared by all five species). Dot size represents the number of genes associated with each term (Count); dot color represents the Benjamini–Hochberg adjusted *p*-value.
